# Evolutionary insights about bacterial GlxRS from whole genome analyses: is GluRS2 a chimera?

**DOI:** 10.1186/1471-2148-14-26

**Published:** 2014-02-12

**Authors:** Saumya Dasgupta, Gautam Basu

**Affiliations:** 1 Department of Biophysics, Bose Institute, P-1/12 CIT Scheme VIIM, Kolkata 700054, India

**Keywords:** GluRS, GluRS2, GlnRS, HGT, tRNA^Gln^, Gene duplication, Phylum-specificity, Whole-genome analysis

## Abstract

**Background:**

Evolutionary histories of glutamyl-tRNA synthetase (GluRS) and glutaminyl-tRNA synthetase (GlnRS) in bacteria are convoluted. After the divergence of eubacteria and eukarya, bacterial GluRS glutamylated both tRNA^Gln^ and tRNA^Glu^ until GlnRS appeared by horizontal gene transfer (HGT) from eukaryotes or a duplicate copy of GluRS (GluRS2) that only glutamylates tRNA^Gln^ appeared. The current understanding is based on limited sequence data and not always compatible with available experimental results. In particular, the origin of GluRS2 is poorly understood.

**Results:**

A large database of bacterial GluRS, GlnRS, tRNA^Gln^ and the trimeric aminoacyl-tRNA-dependent amidotransferase (gatCAB), constructed from whole genomes by functionally annotating and classifying these enzymes according to their mutual presence and absence in the genome, was analyzed. Phylogenetic analyses showed that the catalytic and the anticodon-binding domains of functional GluRS2 (as in *Helicobacter pylori*) were independently acquired from evolutionarily distant hosts by HGT. Non-functional GluRS2 (as in *Thermotoga maritima*), on the other hand, was found to contain an anticodon-binding domain appended to a gene-duplicated catalytic domain. Several genomes were found to possess both GluRS2 and GlnRS, even though they share the common function of aminoacylating tRNA^Gln^. GlnRS was widely distributed among bacterial phyla and although phylogenetic analyses confirmed the origin of most bacterial GlnRS to be through a single HGT from eukarya, many GlnRS sequences also appeared with evolutionarily distant phyla in phylogenetic tree. A GlnRS pseudogene could be identified in *Sorangium cellulosum*.

**Conclusions:**

Our analysis broadens the current understanding of bacterial GlxRS evolution and highlights the idiosyncratic evolution of GluRS2. Specifically we show that: i) GluRS2 is a chimera of mismatching catalytic and anticodon-binding domains, ii) the appearance of GlnRS and GluRS2 in a single bacterial genome indicating that the evolutionary histories of the two enzymes are distinct, iii) GlnRS is more widespread in bacteria than is believed, iv) bacterial GlnRS appeared both by HGT from eukarya and intra-bacterial HGT, v) presence of GlnRS pseudogene shows that many bacteria could not retain the newly acquired eukaryal GlnRS. The functional annotation of GluRS, without recourse to experiments, performed in this work, demonstrates the inherent and unique advantages of using whole genome over isolated sequence databases.

## Background

The presence of glutaminyl-tRNA synthetase (GlnRS) in bacteria is not universal, occurring only in a subset of extant bacteria [1,2]. Many bacteria that do not contain GlnRS possess a non-canonical copy of glutamyl-tRNA synthetase (GluRS), called GluRS2, in addition to the canonical GluRS (renamed GluRS1 to distinguish it from GluRS2) [3]. GluRS2 catalyzes the formation of Gln-tRNA^Gln^ through an indirect route utilizing glutamyl-tRNA^Gln^ amidotransferase (gatCAB) [4,5]. The third and the major group of extant bacteria possess neither GlnRS nor GluRS2. These bacteria synthesize Gln-tRNA^Gln^ utilizing the canonical GluRS and the heterotrimeric amidotransferase gatCAB via the indirect route [6]. The existence of three extant bacterial groups, characterized by the mutually exclusive presence of GlnRS or GluRS2, or, the absence of both, reflects the complex nature of evolutionary history of bacterial GlxRS (Glx stands for Glu and Gln) (Table 1).

**Table 1:** Distribution of GlnRS, GluRS and gatCAB in bacteria whole genomes

**Bacterial phyla**	**GluRS copy**		**1**	**2**	**2**	**1**	**1**
	**GlnRS**		**×**	**×**	**√**	**√**	**√**
	**gatCAB**		**√**	**√**	**√**	**√**	**×**
	**abbr**	**Total**	**Occurrences**				
Acidobacteria	ad	6	3	2	--	1	--
Green non-sulfur	ns	7	6	--	--	1	--
Green sulfur	gs	6	5	--	--	1	--
Deinococcus-Thermus	dt	6	--	--	--	6	--
Hyperthermophilic	ht	18	12	5	--	1	--
Cyanobacteria	cy	7	7	--	--	--	--
Planctomycetes	pl	5	2	--	--	3	--
Verrucomicrobia	ve	4	--	--	--	4	--
Fusobacteria	fu	5	5	--	--	--	--
Bacteroidetes	ba	14	1	--	--	1	12
Spirochaetes	sp	5	4	--	--	1	--
Chlamydiae	ch	6	6	--	--	--	--
Actinobacteria	ac	17	16	--	--	1	--
Tenericutes	te	6	3	--	--	--	3
Firmicutes	fi	28	18	--	--	8	2
Alpha-proteobacteria	α	69	18	45	2	4	--
Epsilon-proteobacteria	ϵ	10	--	4	6	--	--
Delta-proteobacteria	δ	24	1	--	--	23	--
Gamma-proteobacteria	γ	80	--	7	--	28	45
Beta-proteobacteria	β	43	--	--	--	43	--

**Figure 1:**
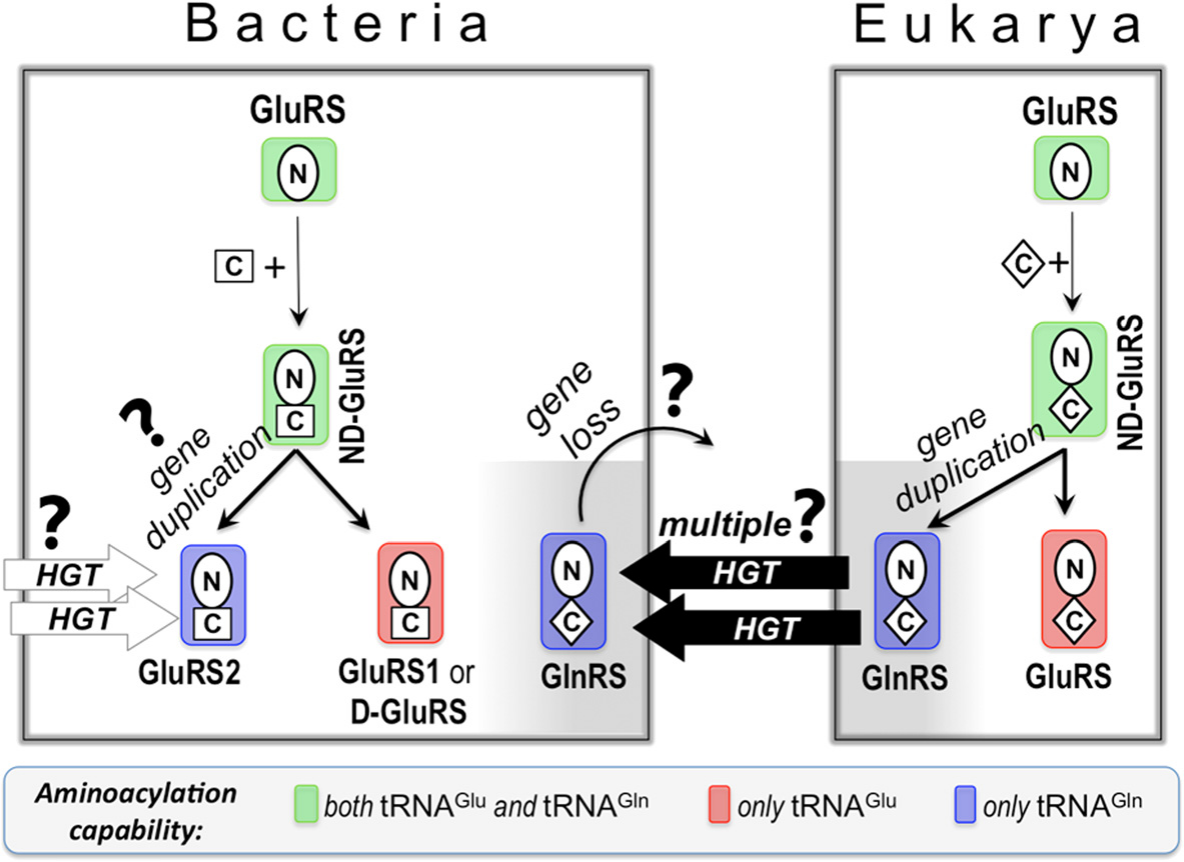
**Evolutionary model of bacterial and eukaryal GlxRS.** The N-terminal catalytic and the C-terminal anticodon-binding domains of GlxRS are annotated by the letters N and C, and depicted according to their mutual homology (oval: N-terminal domains of all GluRS and GlnRS; diamond: C-terminal domains of all GlnRS and eukaryal GluRS; square: C-terminal domains of bacterial GluRS). tRNA^Glx^-aminocylation specificities of GlxRS are indicated by color-coded shades. HGT and ‘?’ stand for horizontal gene transfer and ‘open questions’, respectively.

Although extant GluRS (and GlnRS) is a two-domain protein consisting of a N-terminal catalytic domain and a C-terminal anticodon-binding domain, the C-terminal anticodon-binding domain was added to the catalytic domain only after bacteria and eukaryotes diverged [7-9]. This is reflected in the fact that the anticodon-binding domains of bacterial and eukaryotic GluRS, although functionally similar, are structurally very different (See Figure 1) [10]. GluRS is also considered to be more ancient than GlnRS. GlnRS appeared first in eukaryotes, by gene duplication of GluRS followed by selective amino acid modifications. This is supported by the observation that eukaryotic GluRS and GlnRS in eukaryotes are structurally very similar [11]. However, the same is not true for bacterial GlnRS and GluRS. The anticodon-binding domain of bacterial GlnRS is structurally homologous to eukaryotic GlnRS rather than to bacterial GluRS. Based on this, it has been hypothesized that bacteria acquired GlnRS from eukaryotes by HGT [7,12]. The evolutionary origin of bacterial GluRS2 is not so clear with suggestions that it evolved either from the canonical GluRS/GluRS1 by gene duplication [5] or it appeared in bacteria by HGT [13].

The currently accepted evolutionary history of bacterial GlxRS family, as summarized in Figure 1, is based on insights drawn about two decades ago [7], with later additions [14-17]. Although quite robust in a broad sense, the model needs refinement and re-examination because it is based on GlxRS sequences from only a limited number of bacteria. The weakest point of the model is the poor understanding about the evolutionary origin of GluRS2. Towards this goal, we have compiled and comprehensively analyzed a database consisting of a large number of bacterial whole genomes, taking care to include as many bacterial phyla as possible. Access to whole genomes allowed us not only to analyze sequences of GluRS, GlnRS, gatCAB or tRNA^Glx^, but also to annotate each bacterium and classify them according to the mutual presence or absence of these molecules. Analyses of the resulting annotated whole genome database have yielded new insights about the evolutionary history of bacterial GlxRS. Major findings of the current study can be summarized as: i) GluRS2 is not a gene-duplicated version of GluRS1 but possibly a chimera of evolutionarily distant catalytic and anticodon-binding domains, ii) GlnRS appeared in eubacteria not only by HGT from eukarya but also by intra-bacterial HGT, iii) GlnRS and GluRS2 can coexist in bacterial genomes, iv) identification of a GlnRS pseudo-gene providing direct evidence for the loss of HGT-acquired GlnRS in some bacteria, and v) the importance of nucleotides 32-38 in GlnRS-tRNA^Gln^ coevolution. Our results will help understand the subtleties of a complex molecular coevolution and the database can be used for more insights using complementary techniques.

## Results and discussion

### Bacterial whole genomes classified according to the co-occurrence of GluRS, GlnRS and gatCAB

The availability of a large number of bacterial whole genomes prompted us to revisit the evolutionary history of bacterial GlxRS family. Towards this goal, we constructed a database of bacterial whole genomes, carefully removing redundancies with an attempt to include the widest range of taxonomic lineages (phyla). This resulted in 366 complete bacterial genomes from 16 distinct phyla (Table 1 and Additional files 1 and 2).

A prerequisite for the analysis of sequences present in the database is the classification of bacteria into groups that share a common set of enzymes (among GluRS, GlnRS and gatCAB) for synthesizing Gln-tRNA^Gln^. Although GluRS is present in all bacteria, some possess two copies of the enzyme (GluRS1 and GluRS2) [3]. On the other hand, not all bacteria possess GlnRS or gatCAB. In the light of the above, the database was classified into five groups (see Table 1) according to the presence (+) or absence (-) of GlnRS and gatCAB, and, the number of copies (1 or 2) of GluRS in the genome (the notation has three columns, representing GluRS, GlnRS and gatCAB, respectively) — i) 〈1|-|+〉: GluRS present (one copy), GlnRS absent and gatCAB present, ii) 〈2|-|+〉: GluRS present (two copies), GlnRS absent and gatCAB present, iii) 〈2|+|+〉: GluRS present (two copies), GlnRS present and gatCAB present, iv) 〈1|+|+〉: GluRS present (one copy), GlnRS present and gatCAB present, and, v) 〈1|+|-〉: GluRS present (one copy), GlnRS present and gatCAB absent.

### Distribution of tRNA^Glx^-specificity of GluRS among bacterial phyla

The presence of GluRS is mandatory in all bacteria, whether as a single or as a double copy. In genomes with a single copy of GluRS, the enzyme can be of two functional types, tRNA^Gln^-discriminatory (D-GluRS) and tRNA^Gln^-non-discriminatory (ND-GluRS). Absence of GlnRS in the genome that contains a single copy of GluRS (〈1|-|+〉 in Table 1) indicates ND-GluRS. Presence of GlnRS and the concomitant absence of gatCAB in the genome (〈1|+|-〉 in Table 1) indicates D-GluRS which we term as D^(–)^-GluRS where (-) indicating the absence of gatCAB. Although the tRNA^Glx^-specificity prediction of GluRS for these two groups is robust, the same is not true for the other groups. For example, the concomitant presence of GlnRS as well as gatCAB in the genome (〈1|+|+〉 in Table 1) is not enough information to definitely predict if the GluRS is discriminatory or not. Two GluRSs in 〈1|+|+〉-group (*Thermus thermophilus* and *Pseudomonas aeruginosa*) were experimentally shown to be tRNA^Gln^-discriminatory [18,19]. By extrapolation, we designate GluRSs appearing in the 〈1|+|+〉-group as nominally discriminatory. However, to emphasize that the nomenclature may not be strictly correct, we annotate them as D^(+)^-GluRS. Since genomes with two copies of GluRS also contain gatCAB, a confident guess about the tRNA^Gln^-specificity of GluRS in these bacteria (GluRS1 and GluRS2) is nearly impossible, unless experimentally verified. Earlier, in two such proteobacterial species (*H. pylori* and *Acidithiobacillus ferrooxidans*), the corresponding tRNA^Glx^-specificities of GluRS (GluRS1: tRNA^Glu^-specific and GluRS2: tRNA^Gln^-specific) were experimentally determined [4,5]. We term the two enzymes as GluRS1 (likely to be discriminatory against tRNA^Gln^) and GluRS2 (likely to be discriminatory against tRNA^Glu^). It should be reiterated that although the tRNA^Glx^-specificities of bacterial GluRS, assigned here, are mere predictions, the tRNA^Glx^-specificities of ND-GluRS and D^(-)^-GluRS must match with experimental data due to the absence of co-partners in their respective genomes (gatCAB in case of D^(-)^-GluRS and GlnRS/GluRS2 in case of ND-GluRS); the presence of these co-partners could have made other routes of glutamylation possible.

Table 1 (see Additional files 1 and 2 for details) shows the distribution of the five functional types of GluRS among different bacterial phyla. ND-GluRS is absent in deinococcus-thermus, verrucomicrobia, bacteroidetes (except *Fluviicola taffensis*), δ-proteobacteria (except *S. cellulosum* which, incidentally contains a pseudo gene for GlnRS: see Additional file 1), ϵ-, β- and γ-proteobacteria. On the other hand, ND-GluRS is the only kind of GluRS present in cyanobacteria, fusobacteria and chlamydiae. The cyanobacterial result matches with that of a previous study [20]. D^(–)^-GluRS is present in tenericutes, bacteriodetes (except *Salinibacter ruber*), a few firmicutes and little more than half of all γ-proteobacteria in our database. The single copy of GluRS in all other GlnRS-containing bacteria is D^(–)^-GluRS since their genomes also lack gatCAB. The presence of GluRS2 is restricted to three bacterial phyla — proteobacteria, hyperthermophilic bacteria (5 out of 18) and acidobacteria (2 out of 5). Within the proteobacterial phylum, the presence of GluRS2 is mostly restricted to two classes: ϵ- (all) and α- (47 out of 69), while the occurrence of GluRS2 in other proteobacterial classes is rare, if not absent: γ- (7 out of 80), δ- (none) and β- (none). Overall, GluRS functional types are distributed across all phyla with a clear phylum-specific preference.

### Phylogeny of bacterial GluRS

**Figure 2:**
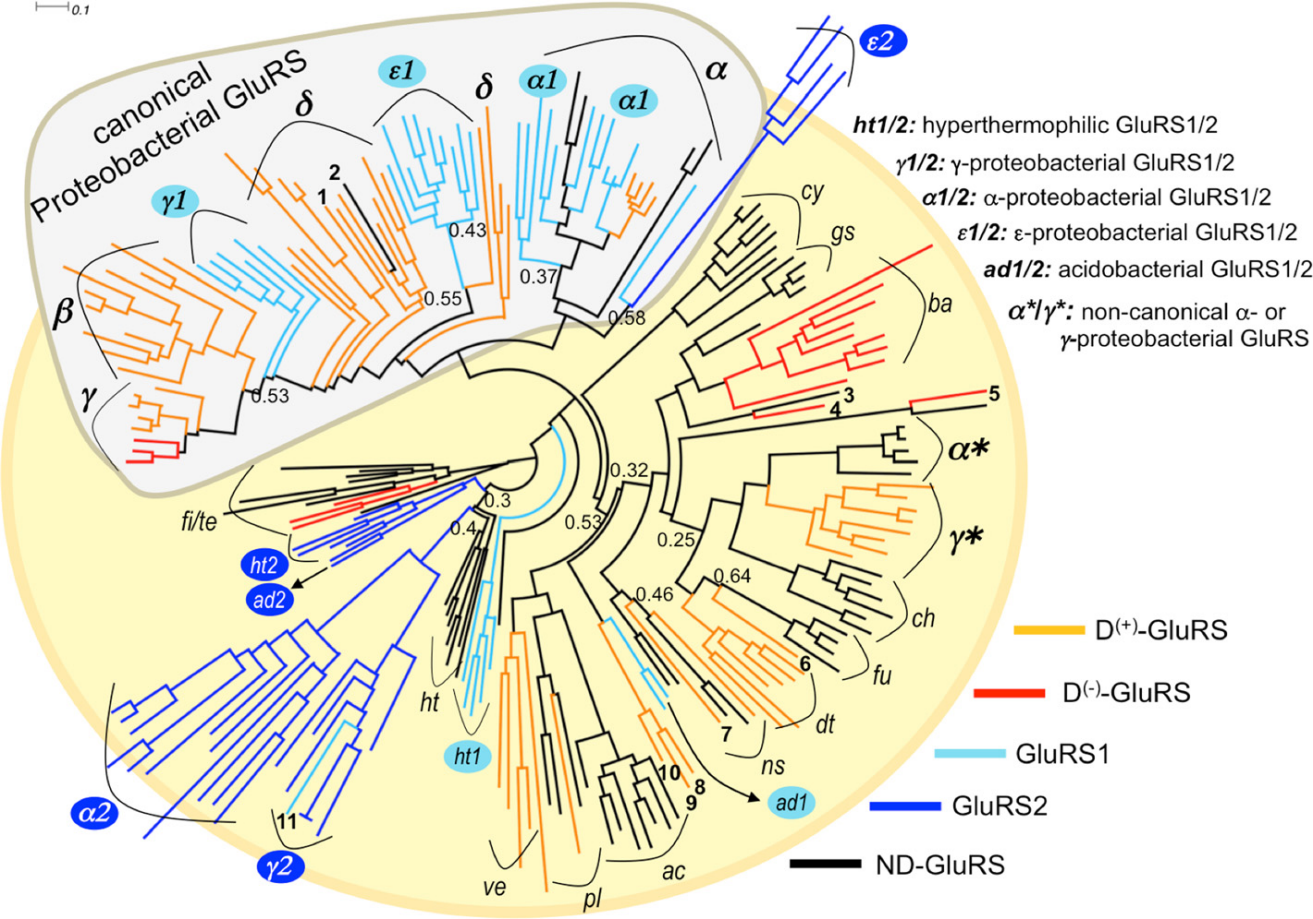
**Phylogeny of bacterial GluRS.** Maximum Likelihood based rooted phylogenetic tree of bacterial GluRS sequences (See Methods). The functional status (see main text) of each GluRS sequences is indicated by a coloring scheme and clades are annotated by abbreviated phylum or class codes (see Table 1). Outliers (three-letter codes given Additional files 1 and 2) for panel are marked by numbers (1: NDE (ht); 2: TID (ht); 3:FMA (fi); 4: AOE (fi); 5:CTH (fi); 6: HOH (δ); 7: SSM (sp); 8: DPR (δ); 9: DPS(δ); 10: DAK (δ); 11: TGR1 (γ)). The canonical proteobacterial group is highlighted along with two groups of outlier γ- and α- proteobacterial GluRS (marked as γ* and α* and listed in Additional file 3). Branch support values < 0.7, using aLRT statistics, are indicated.

**Figure 3:**
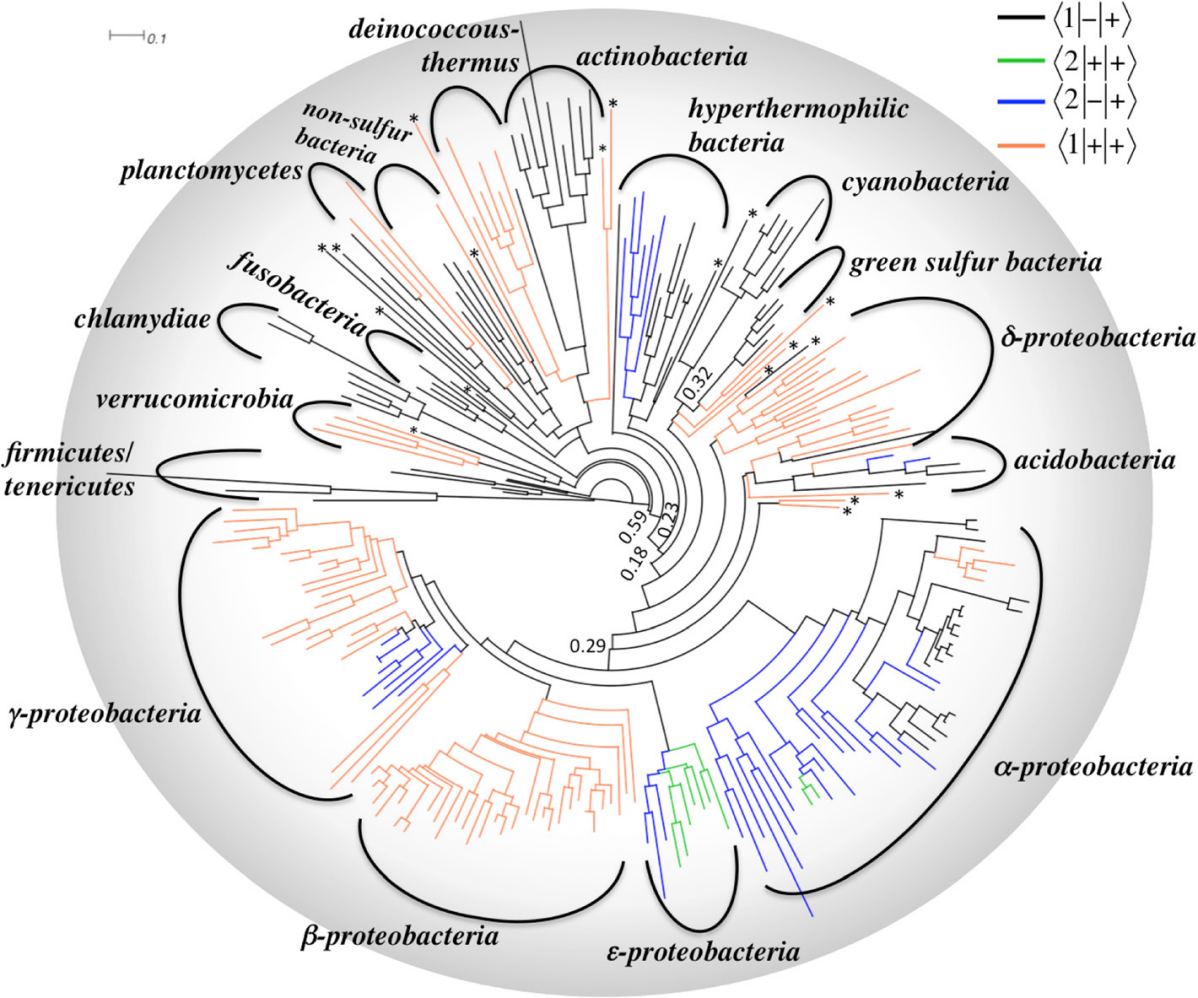
**Phylogenetic tree of bacterial gatB sequences.** Maximum Likelihood based rooted phylogenetic tree of bacterial gatB sequences (See Methods), annotated with bacterial phyla and colored according to the presence or absence of GlnRS and GluRS2 in the genome (see Table 1 for details). The outliers are indicated by an asterisk symbol (clockwise from the root: LIE (sp), MTA (fi); FMA (fi); TPA (sp); BBU (sp); TTR (ht); SSM (sp); SFU (δ); SAT (δ); BPJ (sp); FTE (ba); SRU (ba); BBA (δ); HMR (δ); TID (ht); NDE (ht); PCA (δ) and GLO (δ)). Three-letter bacterium names follow KEGG naming scheme (Additional files 1 and 2). The branch support is calculated using aLRT statistics and only the scores <0.7 are indicated (See Methods).

The phylogenetic tree of representative bacterial GluRS sequences (see Additional file 3) is shown in Figure 2. The tree was constructed from all five functional flavors of GluRS described above. Except GluRS2, majority of proteobacterial GluRSs appear as a separate cluster and is farthest from the root (tenericutes/firmicutes). Non-proteobacterial GluRS also show phylum-specific clustering and the overall branching is compatible with bacterial phylogeny [21]. However, phylum-specific clustering of GluRS is not obeyed by some bacterial species. Two subgroups of γ- and α-proteobacterial GluRS sequences, marked as γ* and α* in Figure 2 and listed in Additional file 4, exhibit non-canonical behavior. These GluRS sequences appear in the non-proteobacterial cluster, as sister clades of chlamydiae, fusobacteria and deinococcous-thermus. Unlike the canonical proteobacterial GluRS (the grey shaded region of Figure 2), GluRS belonging to the γ*-/α*-group seem to have appeared through some alternate evolutionary route, probably via HGT, as has been noted earlier [22]. Interestingly, in gatB phylogeny (Figure 3) the gatB sequences of the γ*-/α*-group are not outliers, indicating that only GluRS and not gatB appeared by HGT in these bacteria. Few δ-proteobacterial GluRS (*Desulfobulbus propionicus*, *Desulfotalea psychrophila*, *Desulfurivibrio alkaliphilus* and *Haliangium ochraceum*) also appear in the non-proteobacterial clades. However, unlike the γ*-/α*-group, gatB sequences of the outlier δ-proteobacteria (in GluRS phylogeny) are also outliers in gatB phylogeny (Figure 3). This behavior could be a result of the atypical genome organizations of δ-proteobacterial species, resulting from their diverse ecologies, metabolic strategies and adaptations, which can facilitate unforeseen HGT events leading to the acquisition of both GluRS and gatB from evolutionarily distant bacterial phyla, or atypical proteins in these bacteria could have resulted from atypical evolutionary pressure [23]. Two non-proteobacterial GluRS, from hyperthermophilic bacteria (*Nitrospira defluvii* and *Thermodesulfatator indicus*), appear in the δ-proteobacterial clade. In addition, there are examples where a non-proteobacterial GluRS appears with other non-proteobacterial GluRS but not within the parent cluster. Overall, although GluRS phylogeny and the whole bacterial phylogeny are more or less consistent, Figure 2 also shows inconsistencies that could be interpreted as the result of systematic (phylum-specific) or occasional HGT among distant eubacteria.

### Correlation between tRNA^Glx^-specificity of GluRS and branching of GluRS/gatB phylogeny

We also probed the evolutionary divergence of the different functional types of GluRS within a given phylum. As shown in Figure 2, D-GluRS and ND-GluRS appear in distinct sister clades in α-proteobacteria (D^(+)^-GluRS versus ND-GluRS), firmicutes/tenericutes (D^(–)^-GluRS versus ND-GluRS) and bacteroidetes (D^(–)^-GluRS versus ND-GluRS). Similarly, D^(+)^-GluRS and D^(–)^-GluRS of γ-proteobacteria and bacteroidetes appear in sister clades. The clade-specific appearance of functionally distinct GluRS within a phylum reflects the function-specific evolutionary pressures they experienced to cope with the presence/absence of other genomic components like GlnRS (between ND- and D-GluRS) and/or gatCAB (between D^(+)^- and D^(–)^- GluRS). We also looked for corresponding function-specific branching of gatB in gatB-phylogenetic tree (Figure 3). The phylogeny shows that gatB sequences of a given phylum, but belonging to different groups defined in Table 1, also appear as sister clade (e.g. γ-proteobacteria: 〈1|+|+〉 and 〈2|-|+〉; ϵ-proteobacteria: 〈2|+|+〉 and 〈2|-|+〉; α-proteobacteria: 〈1|+|+〉, 〈2|+|+〉 and 〈2|-|+〉, hyperthermophilic bacteria: 〈1|-|+〉 and 〈2|-|+〉). This demonstrates how GluRS and gatB coevolved according to their functional requirement of facilitating the indirect route of Gln-tRNA^Gln^ synthesis in some bacteria.

### GluRS2 did not evolve by gene duplication

Among the five different functional types of bacterial GluRS (ND-GluRS, D^(–)^-GluRS, D^(+)^-GluRS, GluRS1 and GluRS2), GluRS2 stands out from the rest in terms of its tRNA^Glx^-specificity. It is the only GluRS that is known to be tRNA^Glu^-discriminatory [4,5]. Like GlnRS, GluRS2 exclusively charges tRNA^Gln^[4,5]. However, the final products are different for the two enzymes — Glu-tRNA^Gln^ for GluRS2 and Gln-tRNA^Gln^ for GlnRS [4,5]. The intimate functional relationship between the two enzymes prompted the proposal that GluRS2 is only a few steps away from evolving into GlnRS [24]. However, it is unclear how and under what circumstances GluRS2 appeared in some bacterial genomes. In this context at least two models have been proposed. Hendrickson *et al.* proposed that GluRS2 was acquired by gene duplication [5] while Nureki *et al.* proposed that the enzyme was acquired through HGT from another bacterial phylum [13].

The phylogenetic placement of GluRS2 in Figure 2 allowed us to address this issue in the context of all other functional types of GluRS. If GluRS2 indeed appeared by gene duplication of GluRS, giving rise to GluRS1 and GluRS2, it is expected that GluRS1 and GluRS2 would appear as sister clades in Figure 2[25]. However, for all double GluRS-containing phyla, GluRS1 and GluRS2 appear in clades that are separated from each other by multiple branching. For example, all GluRS1 in α- and γ-proteobacteria appear within the canonical proteobacterial GluRS branch, while the corresponding GluRS2 appear in non-proteobacterial branches. The only exception is the γ-proteobacterium *Thioalkalivibrio sp.* (marked '11' in the γ2 cluster of Figure 2) for which GluRS1 and GluRS2 appear in sister branches within the γ-proteobacterial GluRS2 cluster. GluRS2 of ϵ-proteobacteria branch out from the canonical GluRS/GluRS1 cluster of α-proteobacteria. Similarly, while GluRS2 of acidobacteria and hyperthermophilic bacteria branch out from firmicutes/tenericutes, the corresponding GluRS1 are evolutionarily distant. Taken together, this indicates that GluRS2 appeared in bacteria by gene acquisition from some foreign host by HGT and not by gene duplication.

### Phylogeny of catalytic and anticodon-binding domains of GluRS

**Figure 4:**
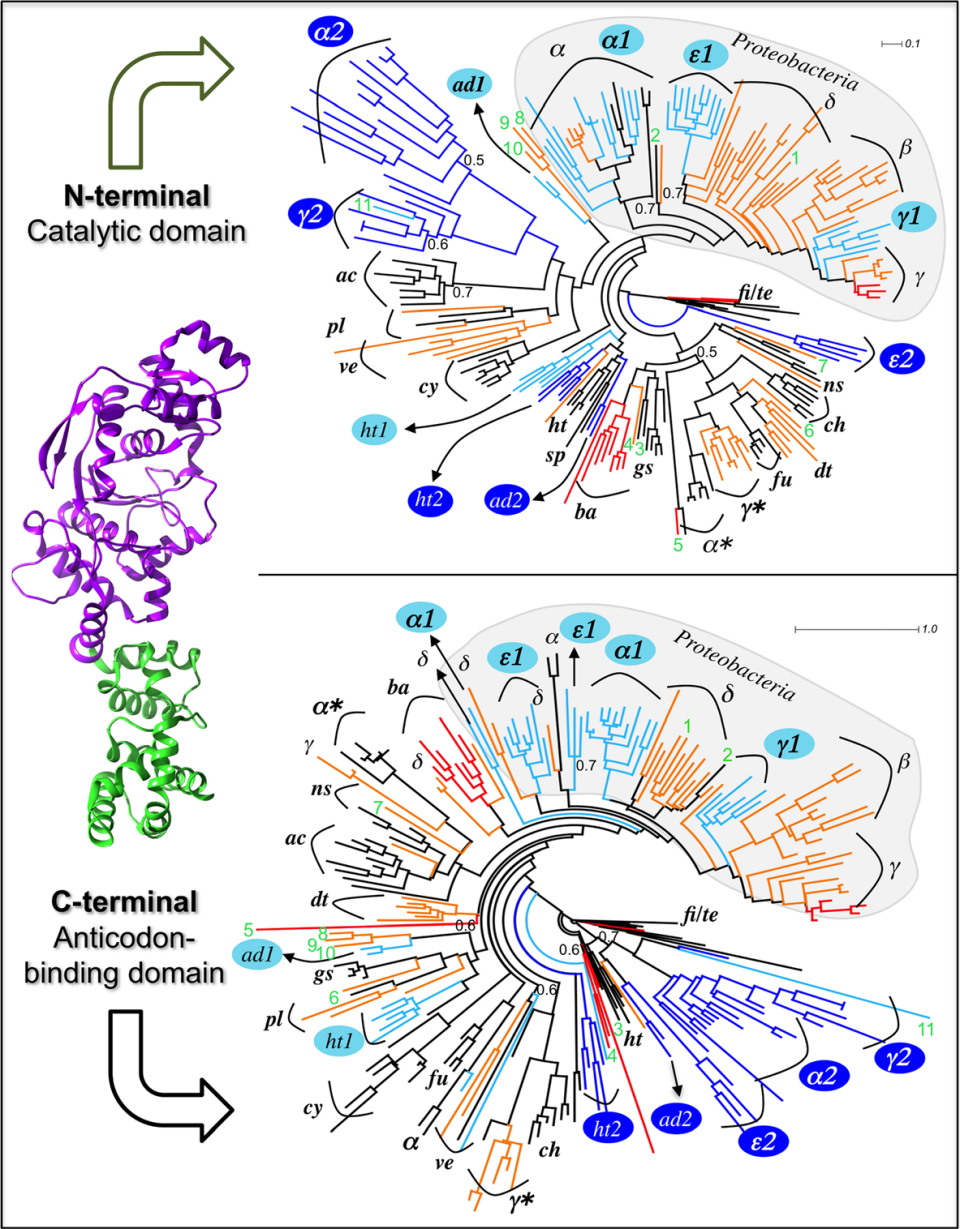
**Phylogeny of N-terminal catalytic and the C-terminal anticodon-binding domains of bacterial GluRS.** All annotations marking the trees are consistent with Figure 2. Branch support values < 0.7, using aLRT statistics, are indicated. The structure shown on the left corresponds to the crystal structure of *T. thermophilus* GluRS (pdb ID: 1j09) with residues 1-322 and 323-468 comprising the N- and the C-terminal domains, respectively.

It is thought that the primordial GluRS consisted of only the N-terminal catalytic domain (GluRS^(N)^). Later, during the course of evolution, the C-terminal domain (GluRS^(C)^) was appended to it [7,12]. As a consequence, the two domains may not display identical branching patterns in phylogenetic trees constructed independently from the two isolated domains. Indeed, a comparison of GluRS^(N)^ and GluRS^(C)^ phylogenies (upper and lowers panels in Figure 4) showed that except for the canonical proteobacterial GluRS group (containing GluRS and GluRS1), the GluRS^(N)^- and GluRS^(C)^-derived cladograms are not strictly mirror images of each other. One reason for this observation could be that GluRS^(C)^ was appended after the phylum-specific divergence of GluRS^(N)^ in bacteria. However, according to this model different bacterial phyla acquired different GluRS^(C)^ independently, which is a very unlikely event. A more realistic model is where GluRS^(C)^ was appended to GluRS^(N)^ before bacterial phylum-divergence but because the acquired GluRS^(C)^ was non-functional, it was lost and regained several times, probably via intra-bacterial HGT, before becoming functionally compatible with GluRS^(N)^ in a synchronous way [26,27]. This model is compatible with Figure 4. In other words, GluRS^(C)^ is more mobile than GluRS^(N)^ and is prone to frequent intra-bacterial HGT. Figure 4 also suggests that GluRS^(N)^ is the core functional domain of GluRS, since the branching topology of GluRS^(N)^ phylogeny (upper panel of Figure 4), but not GluRS^(C)^ phylogeny (lower panel of Figure 4), is compatible with the overall bacterial phylogeny [21].

### Is GluRS2 a chimera?

The mobility of GluRS^(C)^ leads to two possible scenarios concerning the origin of bacterial GluRS that were acquired by HGT – the γ*/α*-group and GluRS2. GluRS belonging to these groups could have been acquired either as a full length GluRS or they appeared by independent acquisition of GluRS^(N)^ and GluRS^(C)^. If the full-length GluRS was acquired then the corresponding GluRS^(N)^ and GluRS^(C)^ are expected to form sister clades with identical GluRS groups in GluRS^(N)^ and GluRS^(C)^ phylogenies (Figure 4). On the other hand, if GluRS^(N)^ and GluRS^(C)^, in GluRS^(N)^ and GluRS^(C)^ phylogenies (Figure 4), were acquired independently then the sister clades of the acquired GluRS^(N)^ and GluRS^(C)^ would be evolutionarily distant and non-identical. For GluRS belonging to the γ*/α*-group, GluRS^(N)^ forms sister clade with the chlamydiae/fusobacteria/deinococcus-thermus/non-green sulphur bacterial group in GluRS^(N)^ phylogeny (Figure 4 upper panel). In GluRS^(C)^ phylogeny (Figure 4 lower panel), GluRS^(C)^ of γ*-group forms a sister clade with GluRS^(C)^ from chlamydiae where as GluRS^(C)^ of α*-group forms a sister clade with GluRS^(C)^ from non-green sulphur bacteria. This suggests that GluRS sequences belonging to the γ*/α*-group were acquired as full-length GluRS.

However, this is not the case with GluRS2. In GluRS^(N)^ phylogeny, γ- and α-proteobacterial GluRS2 appear as sister clade of actinobacterial GluRS, ϵ-proteobacterial GluRS2 appear as sister clade of firmicutes/tenericutes GluRS, acidobacterial GluRS2 appear as sister clade of hyperthermophilic bacterial GluRS, while hyperthermophilic bacterial GluRS2 forms a sister clade with hyperthermophilic bacterial GluRS/GluRS1. The wide distribution of GluRS2^(N)^ in GluRS^(N)^ phylogeny is in stark contrast to the distribution of GluRS2^(C)^ in GluRS^(C)^ phylogeny. For proteobacterial and acidobacterial GluRS2^(C)^ sequences appear together as an outgroup clade. This strongly suggests that GluRS2 sequences were not acquired as full-length GluRS but GluRS2^(N)^ and GluRS2^(C)^ were acquired independently. In other words, GluRS2 is a chimera. This model is not inconceivable since the occurrence of isolated N-terminal domain of GluRS, termed as yadB or Glu-Q-RS, is rampant in bacteria [28-30]. Also, there are other examples of functional proteins that are chimeras [31,32], as has been proposed here for bacterial GluRS2. In fact, it has been argued that sharing of domains is a widespread lineage-specific event among a number of aminoacyl-tRNA synthetases like MetRS, GlyRS, ProRS, HisRS, ValRS and ThrRS [33]. Sometimes domains may even be recruited from non-aminoacyl-tRNA synthetases, like the case of ProRS [33].

Separate phylogenies of GluRS^(N)^ and GluRS^(C)^ also revealed that the evolutionary history of hyperthermophilic bacterial GluRS2 is distinct from other bacterial GluRS2. Unlike GluRS2^(N)^ of other phyla, hyperthermophilic bacterial GluRS2^(N)^ seems to be a gene-duplicated version of GluRS1^(N)^ since GluRS1^(N)^/GluRS2^(N)^/GluRS^(N)^ are monophyletic in GluRS^(N)^ phylogeny. However, in GluRS^(C)^ phylogeny the hyperthermophilic bacterial GluRS1^(C)^/GluRS2^(C)^/GluRS^(C)^ are widely dispersed. This suggests that while GluRS2^(N)^ and GluRS2^(C)^ were independently acquired by most phyla, GluRS2^(N)^ in hyperthermophilic bacteria appeared by a gene duplication event while GluRS2^(C)^ was probably acquired independently by HGT.

Independent evidence supporting gene duplication of GluRS1^(N)^ as the origin of GluRS2^(N)^ of hyperthermophilic bacteria, but not for the case of GluRS2^(N)^ of other bacterial phyla, came from the analysis of the ‘HIGH’ sequence motif, a highly conserved motif present in the N-terminal catalytic domain (as part of the Rossmann fold) of class-I aminoacyl-tRNA synthetases [34,35]. The signature motif is highly conserved in bacterial GluRS sequences (See Additional file 3) as HϕGX (ϕ: I/V/L; X: G/N/S/T/L/M). For majority (159/212) of GluRS^(N)^ sequences used in Figure 4, the motif is HϕGG. This motif is strictly present in GluRS1^(N)^ of α-/ϵ-/γ-proteobacteria and acidobacteria. The corresponding motif in GluRS2^(N)^ of α-/ϵ-/γ-proteobacteria and acidobacteria is HϕGN, suggesting that GluRS2^(N)^ did not appear by gene duplication in these phyla. The ‘HIGH’ motif of α*-/γ*-group of GluRS^(N)^ sequences, HϕGT, is also different from HϕGG, the ‘HIGH’ motif of canonical α-/γ-proteobacterial GluRS. This is consistent with HGT as the origin of α*-/γ*-group of GluRS sequences. In contrast, both GluRS1^(N)^ and GluRS2^(N)^ of hyperthermophilic bacteria share a common ‘HIGH’ motif, HϕGG. This supports the hypothesis that GluRS2^(N)^ is a gene-duplicated version of GluRS1^(N)^ in hyperthermophilic bacteria but not in α-/ϵ-/γ-proteobacteria and acidobacteria.

### Two functionally and evolutionarily distinct types of GluRS2

**Figure 5:**
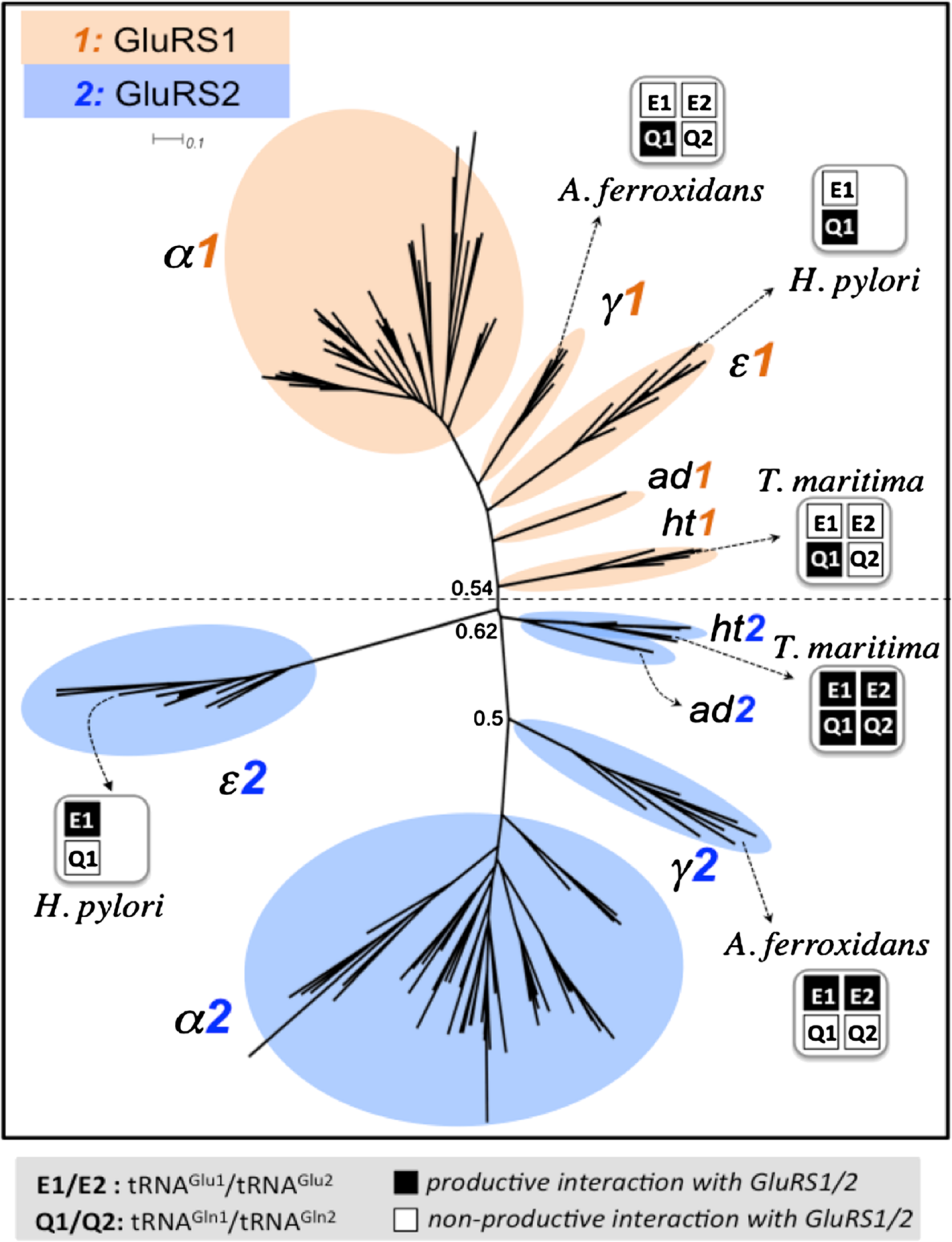
**Phylogeny of bacterial GluRS1 and GluRS2.** Phylogenetic tree of bacterial GluRS1 and GluRS2 sequences (listed in Additional file 5) and annotated with bacterial phyla (abbreviations in Table 1). Experimentally determined glutamylation capacity of both GluRS1 and GluRS2 for selected bacterial species (*H. pylori*, *A. ferrooxidans* and *T. maritima*) with the two isoacceptors of tRNA^Glu^ (E1: ^34^UUC and E2: ^34^CUC) and tRNA^Gln^ (Q1: ^34^UUG and Q2: ^34^CUG), are projected in the respective clades, as productive or non-productive (empty/filled symbols). Branch support values < 0.7, calculated using aLRT statistics, are indicated.

In order to further probe the evolutionary relationship between GluRS1 and GluRS2, a phylogenetic tree was constructed, exclusively with GluRS1 and GluRS2 sequences (Additional file 5). The GluRS1/GluRS2 phylogeny (Figure 5) shows a clear division between GluRS1 and GluRS2 sequences with α-proteobacterial GluRS1 and GluRS2 farthest from each other; the ϵ-proteobacterial GluRS2 appears evolutionary far from the rest. Interestingly, GluRS1/GluRS2 of hyperthermophilic bacteria appear at the border of GluRS1/GluRS2 separation.

Since the tRNA^Glx^-specificities of a number of GluRS1/2 sequences are experimentally known, these are projected onto Figure 5 for further insights. GluRS1 of ϵ-proteobacteria *H. pylori* is tRNA^Glu^-specific; it does not glutamylate the sole tRNA^Gln^ isoacceptor (tRNA^Gln(UUG)^) present in the genome. In a complementary fashion, the corresponding GluRS2 of *H. pylori* glutamylates tRNA^Gln(UUG)^ and not tRNA^Glu^[5]. The tRNA^Gln^-specificity of GluRS1 of γ-proteobacteria *A. ferrooxidans* is isoacceptor-specific – it does not glutamylate tRNA^Gln(UUG)^ but is capable of glutamylating tRNA^Gln(CUG)^. The corresponding GluRS2 glutamylates both isoacceptors, tRNA^Gln(UUG)^ and tRNA^Gln(CUG)^, but none of the two tRNA^Glu^ isoacceptors [4]. The experimental data can be interpreted to indicate that members of the γ-/ϵ-proteobacterial GluRS1-clusters are tRNA^Glu^-specific (discriminatory against tRNA^Gln(UUG)^) while those in the γ-/ϵ-proteobacterial GluRS2-cluster are tRNA^Gln^-specific (discriminatory against tRNA^Glu(UUC/CUC)^). In contrast, the tRNA^Glx^-specificities of gene-duplicated GluRS1/2 of hyperthermophilic bacteria are non-canonical. The GluRS1 of hyperthermophilic bacterium *T. maritima* is experimentally known to be non-specific (it charges both tRNA^Glu^ and tRNA^Gln^) while the corresponding GluRS2 is inactive (it doesn’t charge either tRNA^Glu^ or tRNA^Gln^) [36]. One could generalize this observation as: GluRS2 are inactive while GluRS1 are tRNA^Glx^-non-specific (ND-GluRS).

The functional annotation can be used to predict the tRNA^Glx^-specificity of GluRS1 and GluRS2 of acidobacteria (*Koribacter versatilis* and *Acidobacterium capsulatum*) and for the rest, for which no experimental data are available (Additional file 5). Since acidobacterial GluRS2 appears with GluRS2 of *T. maritima* in Figure 5, taken at face value, acidobacterial GluRS2 should also be inactive. It is interesting to note that GluRS2 of acidobacteria and GluRS2 of hyperthermophilic bacteria appear as sister clades in the master GluRS phylogeny as well (Figure 2). If acidobacterial GluRS2 are indeed inactive, then the corresponding GluRS1 must be tRNA^Gln^-non-discriminatory (ND-GluRS). Since the acidobacterial genomes in our database contain both tRNA^Gln^ isoacceptors (NCBI-GeneID: 4070219^(UUG)^/4068718^(CUG)^ for *Koribacter versatilis* and 7699874^(UUG)^/7698803^(CUG)^ for *Acidobacterium capsulatum*), and the corresponding GluRS1 sequences appear close to the proteobacterial GluRS1-cluster (Figure 5), by extrapolation, we predict that acidobacterial GluRS1 is capable of glutamylating tRNA^Gln(CUG)^ but not tRNA^Gln(UUG)^ (like GluRS1 of *A. ferrooxidans*). This analysis shows that GluRS2 comes in two distinct flavors, both in terms of evolution and function. The first type of GluRS2, appearing by gene-duplication of the N-terminal catalytic domain and later recruitment of an anticodon-binding domain is non-functional (cannot glutamylate tRNA^Glx^). The second type of GluRS2, a chimera of N-terminal catalytic domain and C-terminal anticodon-binding domain, both acquired by HGT is functional and can only glutamylate tRNA^Gln^.

### Distribution of GlnRS among bacterial phyla

It is generally accepted that GlnRS is present mostly in proteobacteria, a phylum of recent divergence. Among non-proteobacteria, some members of deinococcous-thermus [37], firmicutes [4] and bacteroidetes [16] have been reported to possess GlnRS. A survey of our database (Table 1) shows that all members of β- and δ-proteobacteria (except one, *Sorangium cellulosum*, which contains a GlnRS pseudogene) contain GlnRS. Except for seven species (*Acidithiobacillus ferrooxidans*, *Methylococcus capsulatus*, *Alkalilimnicola ehrlichei*, *Halorhodospira halophila*, *Thioalkalivibrio sp*., *Nitrosococcus oceani* and *Coxiella burnetii*), all γ-proteobacteria also contain GlnRS. On the other hand, only six (out of 69) α-proteobacteria, four (*Oligotropha carboxidovorans*, *Nitrobacter hamburgensis*, *Bradyrhizobium japonicum* and *Rhodopseudomonas palustris*) without and two (*Mesorhizobium sp.* and *Mesorhizobium loti*) with GluRS2 in their genomes contain GlnRS. All ten ϵ-proteobacteria in our database contains GluRS2, among which six (*S. denitrificans*, *A. butzleri*, *Sulfuricurvum kujiense*, *Sulfurospirillum deleyianum*, *Sulfurovum sp.* and *Nitratifractor salsuginis*) also contain GlnRS. Among non-proteobacterial phyla, GlnRS is present in deinococcus-thermus (all), bacteroidetes (except *F. taffensis*), planctomycetes (3/5), verrrucomicrobia (all), tenericutes (3 out of 6) and firmicutes (10/28). GlnRS is strictly absent in three non-proteobacterial phyla (fusobacteria, chlamydiae, and cyanobacteria) while the remaining non-proteobacterial phyla contain only a single species whose genome contains GlnRS (Additional files 1 and 2). Thus, GlnRS is widely distributed among bacterial phyla, more than what is currently believed. However, it is mostly present in proteobacteria and a selected group of non-proteobacterial phyla.

### Molecular phylogeny of bacterial GlnRS

**Figure 6:**
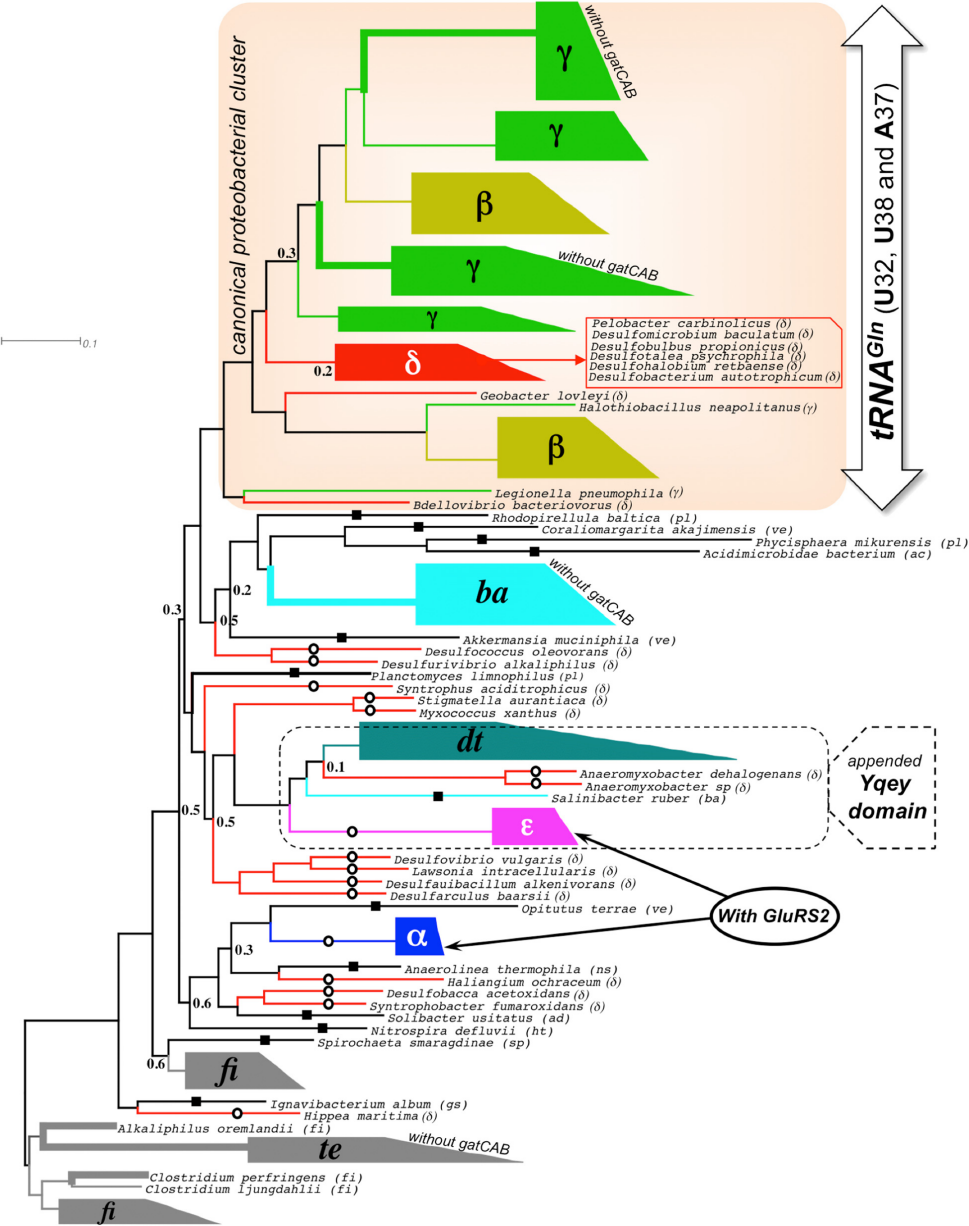
**Phylogeny of bacterial GlnRS.** Phylogenetic tree of bacterial GlnRS sequences, annotated with bacterial phyla or classes (abbreviations in Table 1). Branch support values < 0.7, calculated using aLRT statistics, are indicated. Some GlnRS sequences are highlighted based on the absence or presence of specific features in the GlnRS-containing genome: i) gatCAB-lacking genome (shown by thick lines), ii) GluRS2-containing genome, iii) genomes with Yqey-appended GlnRS, iv) genomes that contain U32-U38-A37 in their tRNA^Gln^. Outlier GlnRS sequences (see text for details) are marked by open circles (proteobacteria) or filled boxes (non-proteobacteria). Selected clades are annotated by phylum names (see Table 1 for abbreviated phylum names).

To gain insight about the origin of GlnRS in eubacteria, a phylogenetic tree was constructed and rooted using the sequences from firmicutes and tenericutes, as out-groups (Figure 6). Bacterial phyla with dominant presence of GlnRS (γ- and β-proteobacteria, firmicutes/tenericutes, bacteroidetes and deinococcus-thermus) cluster in a phylum-specific manner and their branching pattern in the tree is compatible with the overall bacterial phylogeny [21]. This group of GlnRS could have appeared from eukaryotic source by two different routes: i) a single HGT event, or, ii) phylum-specific multiple HGT events. While the second route cannot be ruled out, the overall compatibility of GlnRS phylogeny and bacterial phylogeny suggests that there was a single, and not multiple HGT events, that resulted in the acquisition of eukaryotic GlnRS by bacterium. Subsequently, as bacteria diverged, so did GlnRS, but it could be retained only by some bacterial phyla. Factors that may have played a role in the retention of GlnRS are discussed later.

However, this model cannot fully justify the phylogenetic tree of Figure 6. The placement of a number of GlnRS sequences in the phylogenetic tree is not compatible with the overall bacterial phylogeny. GlnRS from ϵ-proteobacteria, α-proteobacteria and a number of δ-proteobacteria (the exceptions are marked by open circles in Figure 6), do not form sister clades with the canonical proteobacterial GlnRS cluster. GlnRS from ϵ-proteobacteria appear as a sister clade with deinococcus-thermus, while GlnRS from α-proteobacteria appear as a sister clade with a group of isolated non-proteobacteria and δ-proteobacteria. Similarly, non-proteobacterial GlnRS, other than those in firmicutes/tenericutes, bacteroidetes and deinococcus-thermus, are dispersed among proteo- and non-proteobacterial clades (marked by filled square boxes in Figure 6). GlnRS from one bacteroidetes (*S. ruber*; marked by filled square box in Figure 6) is also an outlier. The isolated appearance of GlnRS, distributed across phylum-specific clades, and the appearance of ϵ-, α- and δ-proteobacterial GlnRS, as sister clades of non-proteobacterial GlnRS, suggest intra-bacterial HGT as the origin of these GlnRS, after the initial acquisition of eukaryotic GlnRS in the eubacterial branch.

### Co-occurrence of GluRS2 and GlnRS in the genome

Till date there are no reports of any bacterium possessing both GlnRS and GluRS2. In this context, a remarkable finding is the case of two α- and some ϵ-proteobacteria whose genomes contain both GluRS2 and GlnRS (see Table 1 and Additional file 1). All ten ϵ-proteobacteria in our database contain GluRS2 (and GluRS1), out of which six also contain GlnRS in their genomes. Among the 47 α-proteobacteria whose genomes contain GluRS2 (and GluRS1), only two (genus *Mesorhizobium*) also contain GlnRS. The ϵ-proteobacterial GlnRS and deinococcus-thermus GlnRS appear as sister clades in GlnRS phylogeny (Figure 6) indicating that ϵ-proteobacteria probably acquired their GlnRS by HGT from deinococcus-thermus (more evidence of this HGT is presented later). The two GlnRS from *Mesorhizobium* appear with four other α-proteobacterial GlnRS (whose genomes do not contain GluRS2 but contain GlnRS), as a sister clade with a heterogeneous group of non-proteobacteria and δ-proteobacteria (Figure 6). Because majority of α-proteobacteria do not possess GlnRS, it appears that these six are exceptional cases where GlnRS was acquired by HGT. In an earlier section we had observed that intra-bacterial GlnRS transfer is a common event. This section shows that the event does not depend on whether or not the receiving species already possesses a specialized enzyme for exclusively aminoacylating tRNA^Gln^ (GluRS2). The co-occurrence of GluRS2 and GlnRS also indicates that their evolutionary histories are independent and that GlnRS did not evolve from GluRS2 as has been suggested elsewhere [5].

### Bacterial GlnRS with its C-terminal appended with Yqey paralog appeared in deinococcus-thermus phylum

GlnRS from three bacteria, *Deinococcus radiodurans* and Deinococcus geothermalis from the deinococcus-thermus phylum and *S. ruber* from the bacteroidetes phylum, have been reported to have an extra domain appended at their C-termini [38,39]. This C-terminal extension is actually a paralog of Yqey protein present in the C-terminal end of the gatB subunit of gatCAB [37]. In *D. radiodurans* the Yeqy paralog enhances tRNA^Gln^-affinity of GlnRS [37]. Using multiple sequence alignment, we searched for the presence of the appended Yqey domain in 195 GlnRS sequences in our database. All GlnRS sequences, belonging to deinococcus-thermus phylum in our database, were found to be C-terminal appended with the Yqey paralog (Additional file 6). Except for *S. ruber*, no other GlnRS from bacteroidetes contained the additional domain (the C-terminal appendix of GlnRS from *Flavobacterium johnsoniae*, a bacteroides, is not an Yqey paralog). In addition, GlnRS from all ϵ- and two δ-proteobacteria also contained the Yqey paralog (Additional file 6). Although the GlnRS phylogenetic tree (Figure 6) was constructed without the C-terminal appended Yqey paralog, the Yqey paralog was found to be appended in all GlnRS sequences that formed sister clades with the deinococcus-thermus clade. This suggests that the Yqey domain was first appended to GlnRS in deinococcus-thermus and later the Yqey-appended GlnRS gene was transferred to some ϵ-proteobacteria, two δ-proteobacteria (*Anaeromyxobacter dehalogenans* and *Anaeromyxobacter sp*.) and one bacteroidete (*S. ruber*).

### Functional status of extant GlnRS in bacteria

Extant GlnRS may or may not be functional [15]. One way to annotate their functional status is to look for gatCAB genes in the genome. Absence of gatCAB gene indicates a defunct indirect glutaminylation pathway, implying that the genomic GlnRS is functional, and more importantly essential. Based on the absence gatCAB in the genome (Table 1, Additional files 1 and 2 and Figure 6), GlnRS from all tenericutes and bacteroidetes (except *S. ruber*) were found to be functional. In addition, more than half of all γ-proteobacterial GlnRS (45/74) were also found to be functional. All but three GlnRS-containing firmicutes contained gatCAB, implying that GlnRS in these three species (*Clostridium difficile*, *Clostridium perfringens* and *Alkaliphilus oremlandii*) must also be functional. Of course, the presence of gatCAB does not necessarily mean that the genomic GlnRS is non-functional, as is the case with three bacteria possessing gatCAB (*P. aeruginosa*, *D. radiodurans* and *T. thermophilus*). The GluRS in these three bacteria were experimentally shown to be tRNA^Gln^-discriminatory, implying that the indirect glutaminylation pathway is defunct and that the GlnRS is functional. By extrapolation, we predict that GlnRS of γ-proteobacteria and deinococcous-thermus are functional.

The presence of GlnRS in ϵ-proteobacteria, all possessing GluRS2, is special. The occurrence of GlnRS in these bacteria was found to be random based on the observation that bacteria of the same genus sometimes contained (*Sulfurimonas autotrophica* and *Arcobacter nitrofigilis*) and sometimes did not contain (*Sulfurimonas denitrificans* and *Arcobacter butzleri*) GlnRS. The random occurrence of GlnRS, most probably acquired by intra-bacterial HGT, along with the obligatory presence of GluRS2, possibly indicates that GlnRS in ϵ-proteobacteria are non-functional. Similarly, GlnRS present in lone members non-proteobacterial phyla (Additional file 2), like *Acidimicrobidae bacterium* (actinobacteria), *Ignavibacterium album* (green sulphur bacteria), *Anaerolinea thermophila* (green non-sulphur bacteria) or *N. defluvii* (hyperthermophilic bacteria) may not be functional. Overall, this analysis shows that extant bacterial GlnRS are very diverse and some may not actually be functional. The database compiled in this paper would be useful to identify some borderline and idiosyncratic GlnRS, whose functional status could be the target of future experimental studies.

### GlnRS changed in a phylum-specific manner when adjusting its tRNA^Glx^-specificity

Is the functionally meaningful GlnRS-tRNA^Gln^ coevolution divergent or convergent? Meaning, does the bacterial kingdom use a universal strategy to optimize GlnRS-tRNA^Gln^ interaction? This is an important question since experimentally determined identity nucleotides in tRNA^Glx^ are often projected as universal across the bacterial kingdom [40]. To address this issue we considered the experimentally determined identity elements of E. coli tRNA^Gln^, a set of nucleotides required for the efficient glutaminylation by GlnRS [41]. The identity determinants of the acceptor stem (U1-A72, G2-C71, G3-C70) and the D-stem (G10) are absolutely conserved in tRNA^Gln^ of GlnRS-containing genomes (Additional file 7).

However, the conservation of the anticodon stem-loop nucleotide 32, 38 and 37 (identity elements: U32, U33, C34, U35, G36, A37 and U38) is irregular. As shown in Figure 6, γ-and β-proteobacterial tRNA^Gln^ sequences are always associated with U32-U38 (along with A37) signature, while the combination is nearly absent (present in a few α- and δ-proteobacterial tRNA^Gln1^) among the rest of bacterial tRNA^Gln^ (Additional file 8). Identity of the 32-38 nucleotide pair is known to influence the anticodon loop conformation through unusual bifurcated hydrogen bond formation with functional implications [42]. Specifically it was shown that the U32-U38 combination is not isosteric with any other combination of nucleotides at 32-38 and that this can induce an unusual conformation of the anticodon loop [43,44].

Despite the differences at 32-38 and 37 nucleotide positions in tRNA^Gln^, representative GlnRS from bacterial groups, one with U32-U38 and A37 (*E. coli* a γ-proteobacterial) [41] and the other with C32-A38 and G37 (*T. thermophilus* from deinococcus-thermus) [18], are experimentally known to be functional. Since GlnRS from *E. coli* and *T. thermophilus* are distant in the phylogenetic tree by multiple branching, one can conclude that GlnRS-tRNA^Gln^ coevolved differently in the two bacteria. In other words, coevolution of bacterial GlnRS-tRNA^Gln^ is phylum-specific or divergent, and, the experimentally determined tRNA^Gln^ identity elements for a bacterium in one phylum (γ-protobacteria) may not strictly hold true for another bacterium belonging to a different phylum (deinococcus-thermus). Such phylum-specific trends have been observed experimentally for GluRS-tRNA^Gln^ interaction – a D-GluRS-specific residue (Arg358) in *Thermus thermophilus* GluRS led to a relaxed tRNA^Gln^-discrimination [45] but when the same residue was mutated in *H. pylori* (GluRS1), no such effect was observed [46]. Similarly, for GluRS-tRNA^Glu^ interaction, it was found that a proteobacteria-specific Arg residue (Arg 266 in *E. coli* GluRS) was absolutely essential for glutamylation efficiency of GluRS but the Arg is replaced by mostly Leu in non-proteobacterial GluRS [22].

## Conclusion

By constructing and analyzing a large database of bacterial whole genomes, we have probed the evolution of Gln-tRNA^Gln^ synthesizing molecular machinery. Our approach is unique because of the large database employed and the functional annotation we used, taking advantage of whole genome information. In addition to supporting the broad picture of the currently accepted model for GlxRS evolution (Figure 1), our results bring out some new findings — the most important being the evolutionary origin of GluRS2. We showed that bacterial GluRS2 comes in two flavors, both in terms of evolution and function. The first kind, found in hyperthermophilic bacteria, appeared by gene duplication of the N-terminal catalytic domain and is non-functional. On the other hand, functional GluRS2, found in some proteobacterial classes (α-, ϵ- and γ-), did not appear due to gene duplication. Rather, these are chimeras of catalytic and anticodon-binding domains, acquired independently by HGT. Acidobacterial GluRS2 is predicted to be functionally similar to hyperthermophilic GluRS2. We could identify extant bacteria that contain both GlnRS and GluRS2, pointing out that their evolutionary histories are independent. In addition, a GlnRS pseudo-gene (in *S. cellulosum*) was identified that provided direct evidence of loss of HGT acquired GlnRS. Another important finding is the correlation of nucleotides at 32-38 position of tRNA^Gln^ and the phylogenetic placement of GlnRS, pointing towards GlnRS-tRNA^Gln^ coevolution and the importance of 32-38 nucleotides in GlnRS-tRNA^Gln^ interaction. We showed that bacterial GlnRS are of two types, one acquired from eukaryotes by HGT and the other appearing later by intra-phyla HGT, as exemplified by the Yqey-appended GlnRS in ϵ- and δ-proteobacteria, acquired from deinococcus-thermus. The results presented here highlight many subtleties of evolution of bacterial GlxRS and may be a general feature of some other bacterial proteins as well. The functional status of some borderline and idiosyncratic GlnRS, pointed out in this work, could be the target of future experimental studies. The annotated database could also be analyzed further for idiosyncratic features of bacterial GlxRS evolution not identified here.

## Methods

### Construction of the database

A total of 366 complete bacterial genomes were analyzed from KEGG genome database [January 2013] [47], from 16 distinct taxonomic lineages or phyla (Additional files 1 and 2). Each genome was searched for the presence of GlnRS (gene: *glnS*), GluRS (gene: *gltX*), gatCAB (simultaneous presence of three genes: *gatA*, *gatB*, *gatC*). For GluRS, we also used additional search criterion (glutamyl- and glutaminyl-) and filtered (for example, rejecting sequences representing only the ~ 280-330 long N-terminal catalytic domain) the results for identifying more than one copy of GluRS. In genomes containing two copies of GluRS, GluRS1 and GluRS2 were annotated by comparing with already annotated isoforms (*H. pylori* GluRS1: NCBI-GI 15645104, GluRS2: NCBI-GI 15645267; *A. ferrooxidans* GluRS1: NCBI-GI 198282724, GluRS2: NCBI-GI 198283983; *T. maritima* GluRS1: NCBI-GI 15644618, GluRS2: NCBI-GI 15644103). The 195 bacterial genomes containing GlnRS were further searched for tRNA^Gln1^ (^34^UUG^36^) and tRNA^Gln2^ (^34^CUG^36^) sequences (Additional file 7). The tRNA^Gln^ sequences were double checked with three other genomic tRNA databases, tRNADB-CE 2011 [48], tRNAdb 2009 [49] and GtRNAdb [50], to resolve inconsistencies.

### Multiple sequence alignment

Multiple alignments of gatB and GlnRS sequences in the database were achieved by MUSCLE using default parameters [51]. Multiple sequence alignment of GluRS was performed using PROMALS3D [52], a structure based alignment web-server, with default parameters and seven crystallographic structures of bacterial GluRS (PDB ID: 1j09, 2cfo, 2ja2, 3afh, 2o5r, 4g6z and 4gri). The alignment of 212 representative GluRS sequences, used to construct phylogenetic trees, is provided in the Additional file 3. Multiple alignments of tRNA^Gln^ sequences were performed manually, consistent with the core tRNA structure (for example structure of E. coli tRNA^Gln^; PDB ID: 1gts) and consistent with available tRNA-alignment in the GtRNAdb/tRNAdb 2009 database. The aligned tRNA^Gln^ sequences are given in Additional file 7.

### Definition of GlxRS domains

The N-terminal catalytic domain and the C-terminal anticodon-binding domains of GluRS were defined from multiple aligned GluRS sequences by annotating residues corresponding to 1-322 and 323-468 of *T. thermophilus* GluRS as the N- and C-terminal domains, respectively [10]. The presence of C-terminal appended Yqey-domain in some bacterial GlnRS was ascertained by projecting the Yqey-containing (*D. radiodurans*; PDB ID: 2hz7, residue 710 - 852) [37] and Yqey-lacking (*E. coli*; PDB ID: 1gts, residue 1-673) GlnRS [53] sequences on the multiple-aligned GlnRS sequences.

### Phylogenetic analysis

The phylogenetic analyses of the GluRS (both the full length and of its N-terminal and C-terminal domain), GlnRS and gatB sequences were performed by the Maximum-likelihood method using the web-server http://www.phylogeny.fr[54] using the a la carte mode. PhyML [55] was utilized for tree building while TreeDyn [56] was utilized for tree rendering. Statistical tests for branches in phylogenetic tree were carried out by the approximate likelihood-ratio test (aLRT) with the null hypothesis corresponding to the assumption that the inferred branch has length 0 [57]. Phylogenetic trees were analysed and reconstructed either as rectangular or circular phylogram by the tree-view software Dendroscope [58]. Phylogenetic trees were rooted at the outgroup firmicutes/tenericutes, consistent with the established phylogeny of bacterial domain of life [21].

### Availability of supporting data

The data sets supporting the results of this article are included within the article (and its additional files) and in the Treebase repository, http://treebase.org/treebase-web/search/study/summary.html?id=15306.

## Competing interests

The authors declare that they have no competing interests.

## Authors’ contributions

SD and GB assembled all sequences, performed data analyses and wrote the manuscript. Both authors read and approved the final manuscript.

## Supplementary Material

Additional file 1 List of proteobacterial genomes used to construct the database used in the study. Click here for file

Additional file 2 List of non-proteobacterial genomes used to construct the database used in the study. Click here for file

Additional file 3 **Multiple aligned GluRS sequences used to derive phylogeny of Figure** 2. Click here for file

Additional file 4 Bacteria belonging to clusters γ* and α* in Figure 2**of the main text.** Click here for file

Additional file 5 List of bacteria containing GluRS1 and GluRS2 and NCBI-GI numbers. Click here for file

Additional file 6 Sequence length distribution of bacterial GlnRS. Click here for file

Additional file 7 **Multiple-aligned tRNA**^**Gln **^**sequences from GlnRS-containing bacteria.** Click here for file

Additional file 8 **Identity features of tRNA**^**Gln **^**isotypes (tRNA**^**Gln1 **^**and tRNA**^**Gln2**^**) at the nucleotides 32, 38 and 37 of the anticodon loop in bacterial genomes with GlnRS gene.** Click here for file
