# Supplementary material for: Evolutionary insights about bacterial GlxRS from whole genome analyses: is GluRS2 a chimera?
Source: BMC Evol Biol. 2014 Feb 12;14:26. doi: 10.1186/1471-2148-14-26 (PMC3927822; doi:10.1186/1471-2148-14-26)
Supplement: Additional file 1 — List of proteobacterial genomes used to construct the database used in the study. [file 1471-2148-14-26-S1.pdf]

List of proteobacterial genomes used to construct the database used in the study.

Bacteria names	Tri-letter code (KEGG)	GlnRS | gatCAB
Proteobacteria Class-γ (80)		
1. *Wigglesworthia glossinidia*	WBR	√×
2. *Enterobacter sp*	ENT	√×
3. *Cronobacter sakazakii*	ESA	√×
4. *Citrobacter rodentium*	CRO	√×
5. *Proteus mirabilis*	PMR	√×
6. *Edwardsiella ictaluri*	EIC	√×
7. *Candidatus Hamiltonella defensa*	HDE	√×
8. *Xenorhabdus bovienii*	XBO	√×
9. *Candidatus Riesia pediculicola*	RIP	√×
10. *Rahnella sp. Y9602*	RAH	√×
11. *Candidatus Moranella endobia*	MEN	√×
12. *Haemophilus somnus 129PT*	HSO	√×
13. *Gallibacterium anatis*	GAN	√×
14. *Stenotrophomonas maltophilia*	SML	√×
15. *Pseudoxanthomonas suwonensis*	PSU	√×
16. *Vibrio anguillarum*	VAN	√×
17. *Alteromonas macleodii*	AMK	√×
18. *Ferrimonas balearica*	FBL	√×
19. *Kangiella koreensis*	KKO	√×
20. *Actinobacillus pleuropneumoniae*	APL	√×
21. *Aeromonas hydrophila*	AHA	√×
22. *Baumannia cicadellinicola*	BCI	√×
23. *Buchnera aphidicola*	BUC	√×
24. *Candidatus Blochmannia floridanus*	BFL	√×
25. *Colwellia psychrerythraea*	CPS	√×
26. *Escherichia coli*	ECO	√×
27. *Haemophilus ducreyi*	HDU	√×
28. *Idiomarina loihiensis*	ILO	√×
29. *Mannheimia succiniciproducens*	MSU	√×
30. *Pasteurella multocida*	PMU	√×
31. *Photorhabdus luminescens*	PLU	√×
32. *Photobacterium profundum*	PPR	√×
33. *Pseudoalteromonas atlantica*	PAT	√×
34. *Psychromonas ingrahamii*	PIN	√×
35. *Salmonella enterica*	STT	√×
36. *Shigella dysenteriae*	SDY	√×
37. *Sodalis glossinidius*	SGL	√×
38. *Vibrio parahaemolyticus*	VPA	√×
39. *Xanthomonas oryzae*	XOP	√×
40. *Xylella fastidiosa*	XFA	√×

41. *Yersinia pseudotuberculosis*	**YPI**	√×
42. *Klebsiella pneumoniae*	**KPN**	√×
43. *Dickeya dadantii*	**DDA**	√×
44. *Pantoea ananatis*	**PAM**	√×
45. *Glaciecola sp.*	**GAG**	√√
46. *Methylomonas methanica*	**MMT**	√√
47. *Methylomicrobium alcaliphilum*	**MAH**	√√
48. *Francisella tularensis*	**FTU**	√√
49. *Thioalkalimicrobium cyclicum*	**TCY**	√√
50. *Allochromatium vinosum*	**ALV**	√√
51. *Halothiobacillus neapolitanus*	**HNA**	√√
52. *Tolumonas auensis*	**TAU**	√×
53. *Dichelobacter nodosus*	**DNO**	√√
54. *Candidatus Ruthia magnifica*	**RMA**	√√
55. *Candidatus Vesicomyosocius okutanii*	**VOK**	√√
56. *Legionella pneumophila*	**LPN**	√√
57. *Thiomicrospira crunogena*	**TCX**	√√
58. *Serratia proteamaculans*	**SPE**	√√
59. *Pseudomonas aeruginosa*	**PAE**	√√
60. *Saccharophagus degradans*	**SDE**	√√
61. *Psychrobacter arcticum*	**PAR**	√√
62. *Hahella chejuensis*	**HCH**	√√
63. *Cellvibrio japonicus*	**CJA**	√√
64. *Azotobacter vinelandii*	**AVN**	√√
65. *Acinetobacter sp.*	**ACI**	√√
66. *Moraxella catarrhalis*	**MCT**	√√
67. *Marinobacter aquaeolei*	**MAQ**	√√
68. *Chromohalobacter salexigens*	**CSA**	√√
69. *Halomonas elongata*	**HEL**	√√
70. *Alcanivorax borkumensis*	**ABO**	√√
71. *Teredinibacter turnerae*	**TTU**	√√
72. *Marinomonas sp.*	**MMW**	√√
73. *Acidithiobacillus ferrooxidans*	**AFE**	×√
74. *Methylococcus capsulatus*	**MCA**	×√
75. *Alkalilimnicola ehrlichei*	**AEH**	×√
76. *Halorhodospira halophila*	**HHA**	×√
77. *Thioalkalivibrio sp*	**TGR**	×√
78. *Nitrosococcus oceani*	**NOC**	×√
79. *Coxiella burnetii*	**CBU**	×√
80. *Gamma proteobacterium HdN1*	**GPB**	√√
Proteobacteria Class-β (43)		
Bacteria names	Tri-letter code (KEGG)	GlnRS | gatCAB
1. *Neisseria meningitidis*	**NMC**	√√
2. *Laribacter hongkongensis*	**LHK**	√√

3. *Pseudogulbenkiania sp*	PSE	√√
4. *Burkholderia mallei*	BMA	√√
5. *Polynucleobacter sp*	PNU	√√
6. *Achromobacter xylosoxidans*	AXY	√√
7. *Taylorella equigenitalis*	TEQ	√√
8. *Pusillimonas sp*	PUT	√√
9. *Polaromonas sp*	POL	√√
10. *Acidovorax avenae*	AAV	√√
11. *Verminephrobacter eiseniae*	VEI	√√
12. *Delftia acidovorans*	DAC	√√
13. *Variovorax paradoxus*	VAP	√√
14. *Comamonas testosteroni*	CTT	√√
15. *Alicycliphilus denitrificans*	ADN	√√
16. *Ramlibacter tataouinensis*	RTA	√√
17. *Methylibium petroleiphilum*	MPT	√√
18. *Herminiimonas arsenicoxydans*	HAR	√√
19. *Minibacterium massiliensis*	MMS	√√
20. *Herbaspirillum seropedicae*	HSE	√√
21. *Candidatus Zinderia insecticola*	ZIN	√√
22. *Collimonas fungivorans*	CFU	√√
23. *Leptothrix cholodnii*	LCH	√√
24. *Thiomonas intermedia*	TIN	√√
25. *Nitrosomonas europaea*	NEU	√√
26. *Aromatoleum aromaticum*	EBA	√√
27. *Azoarcus sp*	AZO	√√
28. *Thauera sp*	TMZ	√√
29. *Methylotenera mobilis*	MMB	√√
30. *Methylovorus glucosetrophus*	MEI	√√
31. *Accumulibacter phosphatis*	APP	√√
32. *Sideroxydans lithotrophicus*	SLT	√√
33. *Gallionella capsiferriformans*	GCA	√√
34. *Bordetella bronchiseptica*	BBR	√√
35. *Chromobacterium violaceum*	CVI	√√
36. *Dechloromonas aromatica*	DAR	√√
37. *Methylobacillus flagellatus*	MFA	√√
38. *Nitrosospira multiformis*	NMU	√√
39. *Ralstonia solanacearum*	RSO	√√
40. *Rhodoferax ferrireducens*	RFR	√√
41. *Thiobacillus denitrificans*	TBD	√√
42. *Variovorax paradoxus EPS*	VPE	√√
43. *Cupriavidus metallidurans*	RME	√√
Proteobacteria Class-δ (24)		
Bacteria names	Tri-letter code (KEGG)	GlnRS | gatCAB
1. *Lawsonia intracellularis*	LIP	√√
2. *Anaeromyxobacter sp*	AFW	√√

3. *Desulfovibrio vulgaris*	DVU	√√
4. *Myxococcus xanthus*	MXA	√√
5. *Pelobacter carbinolicus*	PCA	√√
6. *Anaeromyxobacter dehalogenans*	ADE	√√
7. *Geobacter lovleyi*	GLO	√√
8. *Hippea maritima*	HMR	√√
9. *Desulfarculus baarsii*	DBR	√√
10. *Syntrophobacter fumaroxidans*	SFU	√√
11. *Haliangium ochraceum*	HOH	√√
12. *Stigmatella aurantiaca*	SUR	√√
13. *Syntrophus aciditrophicus*	SAT	√√
14. *Desulfobacca acetoxidans*	DAO	√√
15. *Desulfobacterium autotrophicum*	DAT	√√
16. *Bdellovibrio bacteriovorus*	BBA	√√
17. *Desulfohalobium retbaense*	DRT	√√
18. *Desulfotalea psychrophila*	DPS	√√
19. *Desulfurivibrio alkaliphilus*	DAK	√√
20. *Desulfobulbus propionicus*	DPR	√√
21. *Candidatus Desulfococcus oleovorans*	DOL	√√
22. *Desulfauibacillum Alkenivorans*	DAL	√√
23. *Desulfomicrobium baculatum*	DBA	√√
24. *Sorangium cellulosum*	SCL	×√ (GlnRS pseudogene)
***Proteobacteria Class-α* (69)**		
Bacteria names	Tri-letter code (KEGG)	GlnRS | gatCAB
1. *Oligotropha carboxidovorans*	OCA	√√
2. *Nitrobacter hamburgensis*	NHA	√√
3. *Bradyrhizobium japonicum*	BJA	√√
4. *Rhodopseudomonas palustris*	RPD	√√
5. *Asticcacaulis excentricus*	AEX	×√
6. *Sinorhizobium medicae*	SMD	×√
7. *Agrobacterium tumefaciens*	ATU	×√
8. *Rhizobium etli*	RET	×√
9. *Candidatus Liberibacter asiaticus*	LAS	×√
10. *Caulobacter crescentus*	CCR	×√
11. *Phenylobacterium zucineum*	PZU	×√
12. *Pelagibacter ubique*	PUB	×√
13. *Candidatus Hodgkinia cicadicola*	HCI	×√
14. *Candidatus Midichloria mitochondrii*	MMN	×√
15. *Mesorhizobium sp. BNC1*	MES	√√
16. *Starkeya novella*	SNO	×√
17. *Hyphomicrobium denitrificans*	HDN	×√
18. *Rhodomicrobium vannielii*	RVA	×√
19. *Pelagibacterium halotolerans*	PHL	×√

20. *Brevundimonas subvibrioides*	BSB	×√
21. *Silicibacter pomeroyi*	SIT	×√
22. *Ketogulonicigenium vulgare*	KVU	×√
23. *Sphingobium japonicum*	SJP	×√
24. *Erythrobacter litoralis*	ELI	×√
25. *Gluconobacter oxydans*	GOX	×√
26. *Parvularcula bermudensis*	PBR	×√
27. *Acidiphilium cryptum*	ACR	×√
28. *Brucella abortus*	BMC	×√
29. *Ehrlichia chaffeensis*	ECH	×√
30. *Maricaulis maris*	MMR	×√
31. *Orientia tsutsugamushi Boryong*	OTS	×√
32. *Paracoccus denitrificans*	PDE	×√
33. *Rhodobacter sphaeroides*	RSP	×√
34. *Mesorhizobium loti*	MLO	√√
35. *Neorickettsia sennetsu*	NSE	×√
36. *Novosphingobium aromaticivorans*	NAR	×√
37. *Rhodospirillum rubrum*	RRU	×√
38. *Rickettsia prowazekii*	RPR	×√
39. *Anaplasma phagocytophilum HZ*	APH	×√
40. *Azorhizobium caulinodans*	AZC	×√
41. *Bartonella quintana*	BQU	×√
42. *Hyphomonas neptunium*	HNE	×√
43. *Gluconacetobacter diazotrophicus*	GDI	×√
44. *Ochrobactrum anthropi*	OAN	×√
45. *Beijerinckia indica*	BID	×√
46. *Zymomonas mobilis subsp.*	ZMO	×√
47. *Roseobacter denitrificans*	RDE	×√
48. *Sphingomonas wittichii*	SWI	×√
49. *Magnetospirillum magneticum*	MAG	×√
50. *Dinoroseobacter shibae*	DSH	×√
51. *Granulibacter bethesdensis*	GBE	×√
52. *Jannaschia sp.*	JAN	×√
53. *Xanthobacter autotrophicus*	XAU	×√
54. *Wolbachia pipientis*	WPI	×√
55. *Parvibaculum lavamentivorans*	PLA	×√
56. *Methylobacterium chloromethanicum*	MCH	×√
57. *Methylocella silvestris*	MSL	×√
58. *Hirschia baltica*	HBA	×√
59. *Sphingopyxis alaskensis*	SAL	×√
60. *Azospirillum sp. B510*	AZL	×√
61. *Sinorhizobium fredii USDA 257*	SFD	×√
62. *Agrobacterium radiobacter K84*	ARA	×√
63. *Agrobacterium vitis S4*	AVI	×√
64. *Agrobacterium sp. H13-3*	AGR	×√

65. *Rhizobium leguminosarum bv. viciae*	RLE	×√
66. *Candidatus Liberibacter solanacearum*	LSO	×√
67. *Caulobacter sp. K31*	CAK	×√
68. *Caulobacter segnis*	CSE	×√
69. *Alpha proteobacterium*	APM	×√
Proteobacteria Class-ε **(10)**		
Bacteria names	Tri-letter code (KEGG)	GlnRS | gatCAB
1. *Sulfurimonas denitrificans*	SUA	√√
2. *Campylobacter jejuni*	CJR	×√
3. *Helicobacter pylori*	HPY	×√
4. *Wolinella succinogenes*	WSU	×√
5. *Arcobacter butzleri*	ANT	√√
6. *Nitratiruptor sp.*	NIS	×√
7. *Sulfuricurvum kujiense*	SKU	√√
8. *Sulfurospirillum deleyianum*	SDL	√√
9. *Sulfurovum sp.*	SUN	√√
10. *Nitratifractor salsuginis*	NSA	√√

Each bacterial species is abbreviated by a three-letter code consistent with the KEGG genomic database. The presence or absence of GlnRS and gatCAB is indicated by '√' and '×' respectively. Bacterial genomes with two GluRS genes are highlighted in grey.
