# Supplementary material for: Evolutionary insights about bacterial GlxRS from whole genome analyses: is GluRS2 a chimera?
Source: BMC Evol Biol. 2014 Feb 12;14:26. doi: 10.1186/1471-2148-14-26 (PMC3927822; doi:10.1186/1471-2148-14-26)
Supplement: Additional file 2 — List of non-proteobacterial genomes used to construct the database used in the study. [file 1471-2148-14-26-S2.pdf]

List of non-proteobacterial genomes used to construct the database used in the study.

Spirochaetes (5)

Bacteria names	Tri-letter code (KEGG)	GlnRS | gatCAB
Borrelia burgdorferi	**BBU**	×√
Treponema pallidum	**TPA**	×√
Spirochaeta smaragdinae	**SSM**	√√
Leptospira interrogans serovar	**LIE**	×√
Brachyspira pilosicoli	**BPJ**	×√

Acidobacteria (6)

Bacteria names	Tri-letter code (KEGG)	GlnRS | gatCAB
Granulicella mallensis	**GMA**	×√
Terriglobus saanensis	**TSA**	×√
Candidatus Solibacter usitatus	**SUS**	√√
Chloracidobacterium thermophilum	**CTM**	×√
Koribacter versatilis	**ABA**	×√
Acidobacterium capsulatum	**ACA**	×√

Fusobacteria (5)

Bacteria names	Tri-letter code (KEGG)	GlnRS | gatCAB
Leptotrichia buccalis	**LBA**	×√
Sebaldella termitidis	**STR**	×√
Streptobacillus moniliformis	**SMF**	×√
Ilyobacter polytropus	**IPO**	×√
Fusobacterium nucleatum	**FNU**	×√

Verrucomicrobia (4)

Bacteria names	Tri-letter code (KEGG)	GlnRS | gatCAB
Opitutus terrae	***OTE***	√√
Coraliomargarita akajimensis	**CAA**	√√
Methylacidiphilum infernorum	**MIN**	√√
Akkermansia muciniphila	**AMU**	√√

Planctomycetes (5)

Bacteria names	Tri-letter code (KEGG)	GlnRS | gatCAB
Pirellula staleyi	**PSL**	×√

Planctomyces limnophilus	**PLM**	√√
Isosphaera pallida	**IPA**	×√
Phycisphaera mikurensis	**PHM**	√√
Rhodopirellula baltica	**RBA**	√√
Green sulfur bacteria (6)		
Bacteria names	Tri-letter code (KEGG)	GlnRS | gatCAB
Chlorobaculum tepidum	**CTE**	×√
Chlorobium limicola	**CLI**	×√
Pelodictyon luteolum	**PLT**	×√
Prosthecochloris aestuarii	**PAA**	×√
Chloroherpeton thalassium	**CTS**	×√
Ignavibacterium album	**IAL**	√√
Green non-sulfur bacteria (7)		
Bacteria names	Tri-letter code (KEGG)	GlnRS | gatCAB
Dehalogenimonas lykanthroporepellens	**DLY**	×√
Roseiflexus castenholzii	**RCA**	×√
Chloroflexus aggregans	**CAG**	×√
Herpetosiphon aurantiacus	**HAU**	×√
Anaerolinea thermophila	**ATM**	√√
Thermomicrobium roseum	**TRO**	×√
Dehalococcoides ethenogenes	**DET**	×√
Firmicutes (28)		
Bacteria names	Tri-letter code (KEGG)	GlnRS | gatCAB
Bacillus subtilis	**BSS**	×√
Finegoldia magna	**FMA**	×√
Enterococcus faecalis	**EFA**	×√
Listeria monocytogenes	**LMF**	×√
Lactobacillus gasseri	**LGA**	×√
Streptococcus pneumoniae	**SPN**	×√
Oenococcus oeni	**OOE**	×√
Oceanobacillus iheyensis	**OIH**	×√
Pediococcus pentosaceus	**PPE**	×√
Staphylococcus aureus	**SAH**	×√
Caldicellulosiruptor saccharolyticus	**CSC**	×√
Moorella thermoacetica	**MTA**	×√
Thermoanaerobacter	**TEX**	×√
Lactococcus lactis subsp.	**LLA**	×√
Leuconostoc citreum	**LCI**	×√

Aerococcus urinae	**AUR**	×√
Melissococcus plutonius	**MPS**	×√
Weissella koreensis	**WKO**	×√
Clostridium thermocellum	**CTH**	√×
Alkaliphilus oremlandii	**AOE**	√×
Clostridium clariflavum	**CCL**	√√
Desulfotomaculum reducens	**DRM**	√√
Desulfotomaculum ruminis	**DRU**	√√
Clostridium perfringens	**CPE**	√√
Clostridium ljungdahlii	**CLJ**	√√
Bacillus pseudofirmus	**BPF**	√√
Bacillus cellulosilyticus	**BCO**	√√
Bacillus selenitireducens	BSE	√√

Tenericutes (6)

Bacteria names	Tri-letter code (KEGG)	GlnRS | gatCAB
Mycoplasma pneumoniae	**MPN**	×√
Ureaplasma urealyticum serovar	**UUE**	×√
Mesoplasma florum	**MFL**	×√
Acholeplasma laidlawii	**ACL**	√×
Candidatus Phytoplasma mali	**PML**	√×
Phytoplasma OY	**POY**	√×

Deinococcous-Thermus (6)

Bacteria names	Tri-letter code (KEGG)	GlnRS | gatCAB
Thermus thermophilus	**TTH**	√√
Deinococcus radiodurans	**DRA**	√√
Meiothermus ruber	**MRB**	√√
Truepera radiovictrix	**TRA**	√√
Oceanithermus profundus	**OPR**	√√
Marinithermus hydrothermalis	**MHD**	√√

Bacteroidetes (14)

Bacteria names	Tri-letter code (KEGG)	GlnRS | gatCAB
Salinibacter ruber	**SRU**	√√
Gramella forsetii	**GFO**	√×
Flavobacterium johnsoniae	**FJO**	√×
Porphyromonas gingivalis	**PGI**	√×
Bacteroides fragilis	**BFR**	√×
Parabacteroides distasonis	**PDI**	√×
Candidatus Sulcia muelleri	**SMG**	√×

Capnocytophaga ochracea	**COC**	√×
Riemerella anatipestifer	**RAN**	√×
Paludibacter propionicigenes	**PPN**	√×
Blattabacterium sp.	**BBL**	√×
Cellulophaga algicola	**CAO**	√×
Zobellia galactanivorans	**ZGA**	√×
Fluviicola taffensis	**FTE**	×√

Hyperthermophilic (18)

Bacteria names	Tri-letter code (KEGG)	GlnRS | gatCAB
Candidatus Nitrospira defluvii	**NDE**	√√
Thermobaculum terrenum	**TTR**	×√
Deferribacter desulfuricans	**DDF**	×√
Thermodesulfatator indicus	**TID**	×√
Dictyoglomus thermophilum	**DTH**	×√
Leptospirillum ferrooxidans	**LFC**	×√
Fervidobacterium nodosum	**FNO**	×√
Kosmotoga olearia	**KOL**	×√
Petrotoga mobilis	**PMO**	×√
Thermosipho africanus	**TAF**	×√
Thermotoga maritima	**TMA**	×√
Persephonella marina	**PMX**	×√
Thermocrinis albus	**TAL**	×√
Hydrogenobacter thermophilus	**HTH**	×√
Sulfurihydrogenibium azorense	**SAF**	×√
Aquifex aeolicus	**AAE**	×√
Thermovibrio ammonificans	**TAM**	×√
Desulfurobacterium thermolithotrophum	**DTE**	×√

Cyanobacteria (7)

Bacteria names	Tri-letter code (KEGG)	GlnRS | gatCAB
Prochlorococcus marinus	**PMC**	×√
Microcystis aeruginosa	**MAR**	×√
Synechococcus elongatus	**SYF**	×√
Thermosynechococcus elongatus	**TEL**	×√
Cyanothece sp.	**CYT**	×√
Nostoc punctiforme	**NPU**	×√
Anabaena azollae	**NAZ**	×√

Chlamydia (6)

Bacteria names	Tri-letter code (KEGG)	GlnRS | gatCAB

Chlamydia trachomatis	**CTR**	×√
Chlamydophila pneumoniae	**CPA**	×√
Candidatus Protochlamydia amoebophila	**PCU**	×√
Parachlamydia acanthamoebae	**PUV**	×√
Waddlia chondrophila	**WCH**	×√
Simkania negevensis	**SNG**	×√
Actinobacteria (17)		
Bacteria names	Tri-letter code (KEGG)	GlnRS | gatCAB
Rhodococcus erythropolis	**RER**	×√
Acidimicrobidae bacterium	**AYM**	√√
Streptomyces coelicolor	**SCO**	×√
Renibacterium salmoninarum	**RSA**	×√
Micrococcus luteus	**MLU**	×√
Geodermatophilus obscurus	**GOB**	×√
Bifidobacterium adolescentis	**BAD**	×√
Corynebacterium glutamicum	**CGL**	×√
Clavibacter michiganensis	**CMS**	×√
Mycobacterium tuberculosis	**MTU**	×√
Salinispora tropica	**STP**	×√
Rubrobacter xylanophilus	**RXY**	×√
Tropheryma whipplei	**TWS**	×√
Frankia sp.	**FRA**	×√
Arthrobacter sp.	**ART**	×√
Kocuria rhizophila	**KRH**	×√
Rothia mucilaginosa	**RMU**	×√

Each bacterial species is abbreviated by a three-letter code consistent with the KEGG genomic database. The presence or absence of GlnRS and gatCAB is indicated by '√' and '×' respectively. Bacterial genomes with two GluRS genes are highlighted in grey.
