# Supplementary material for: Evolutionary insights about bacterial GlxRS from whole genome analyses: is GluRS2 a chimera?
Source: BMC Evol Biol. 2014 Feb 12;14:26. doi: 10.1186/1471-2148-14-26 (PMC3927822; doi:10.1186/1471-2148-14-26)
Supplement: Additional file 3 — Multiple aligned GluRS sequences used to derive phylogeny of Figure 2. [file 1471-2148-14-26-S3.pdf]

Additional File-3

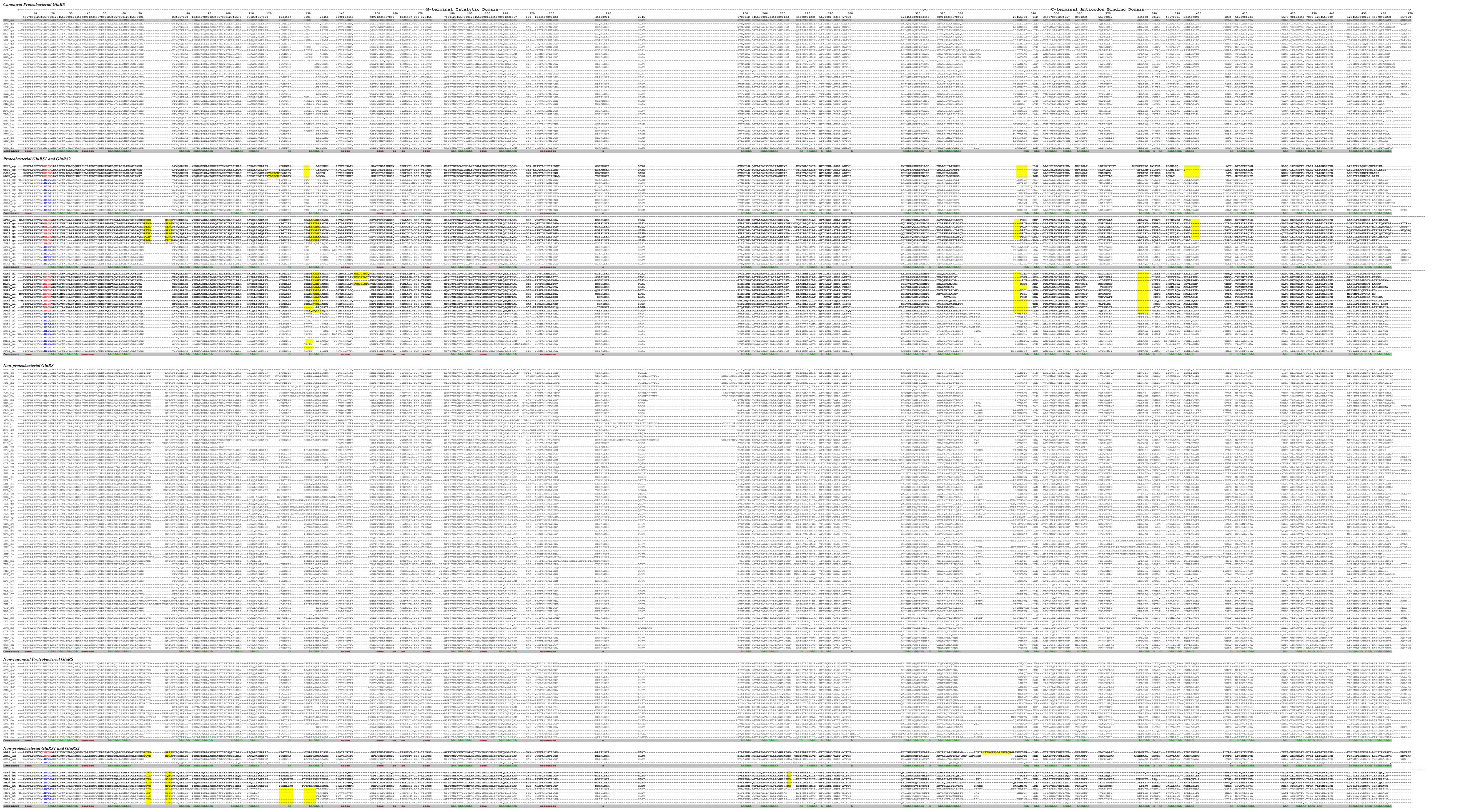

Multiple Sequence alignment of bacterial GluRS by PROMALS3D [50], using the GluRS crystallographic structures (PDB 1j09, 2cfo, 2ja2, 2o5r, 3afh, 4g6z and 4gri) (See Materials and Methods).
Each sequence is annotated with the corresponding species name (tri-letter code) and the phylum/class codes (See Table 1, Additional File 1/2 for details).
The GluRS1 and GluRS2 sequences are marked by 1 and 2 respectively, followed by the tri-letter codes. Non-canonical proteobacterial groups γ* and α* are shown as ga* and al*.
Proteobacterial classes are further annotated with the following codes: ga: class-γ, ep: class-ε; be: class-β; al: class-α, de: class-δ.
The consensus structural folds of the alignment file (obtained from PROMALS3D) are indicated with the anotation 'h': helix and 'e': beta strand.
Sequence insertions/deletions of the respective N-terminal catalytic domain and C-terminal anticodon-binding domain are highlighted. Sequence insertions/deletions of the respective N-terminal catalytic domain and C-terminal anticodon-binding domain are highlighted.
