# Supplementary material for: Evolutionary insights about bacterial GlxRS from whole genome analyses: is GluRS2 a chimera?
Source: BMC Evol Biol. 2014 Feb 12;14:26. doi: 10.1186/1471-2148-14-26 (PMC3927822; doi:10.1186/1471-2148-14-26)
Supplement: Additional file 4 — Bacteria belonging to clusters γ* and α* in Figure 2of the main text. [file 1471-2148-14-26-S4.pdf]

Bacteria belonging to clusters $\gamma*$ and $\alpha*$ in Fig. 2 of the main text.

Cluster	***Bacterial species***
γ*	*Acinetobacter sp.*<br>*Alcanivorax borkumensis*<br>*Azotobacter vinelandii*<br>*Cellvibrio japonicus*<br>*Chromohalobacter salexigens*<br>*Francisella tularensis*<br>*Gamma proteobacterium HdN1*<br>*Hahella chejuensis*<br>*Halomonas elongata*<br>*Marinobacter aquaeolei*<br>*Marinomonas sp.*<br>*Moraxella catarrhalis*<br>*Pseudomonas aeruginosa*<br>*Psychrobacter arcticum*<br>*Saccharophagus degradans*<br>*Teredinibacter turnerae subsp. Tularensis TI0902*
α*	*Agrobacterium radiobacter K84*<br>*Agrobacterium sp. H13-3*<br>*Agrobacterium tumefaciens*<br>*Agrobacterium vitis S4*<br>*Asticcacaulis excentricus*<br>*Candidatus Liberibacter asiaticus*<br>*Candidatus Liberibacter solanacearum*<br>*Rhizobium etli*<br>*Rhizobium leguminosarum bv. viciae*<br>*Sinorhizobium fredii USDA 257*<br>*Sinorhizobium medicae*
