# Supplementary material for: Evolutionary insights about bacterial GlxRS from whole genome analyses: is GluRS2 a chimera?
Source: BMC Evol Biol. 2014 Feb 12;14:26. doi: 10.1186/1471-2148-14-26 (PMC3927822; doi:10.1186/1471-2148-14-26)
Supplement: Additional file 5 — List of bacteria containing GluRS1 and GluRS2 and NCBI-GI numbers. [file 1471-2148-14-26-S5.pdf]

Bacteria containing GluRS1 and GluRS2 with NCBI-GI numbers. *

KEGG code (phylum)	GluRS1	GluRS2	KEGG code (phylum)	GluRS1	GluRS2
HNE (α)	114798943	114799057	AFE (γ)	198282724 (E1/E2/Q2)	198283983 (Q1/Q2)
GDI (α)	162145889	162147995	MCA (γ)	53804443	53805249
OAN (α)	153009373	153009428	AEH (γ)	114320458	114320962
BID (α)	182678393	182680569	HHA (γ)	121998064	121996917
ZMO (α)	56552860	56551796	TGR (γ)	220934111	220934751
RDE (α)	110680399	110678547	NOC (γ)	77163799	77165713
SWI (α)	148556116	148555354	CBU (γ)	215918912	215919194
MAG (α)	83311902	83310334	MMN (α)	339320003	339320233
DSH (α)	159044354	159045288	MES (α)	110633997	110633888
GBE (α)	114327478	114328080	SNO (α)	298291808	298290918
JAN (α)	89054333	89056127	HDN (α)	300023414	300023352
XAU (α)	154248289	154248640	RVA (α)	312115925	312115465
WPI (α)	190571023	190570625	PHL (α)	357384398	357383948
PLA (α)	154253619	154251558	BSB (α)	302383256	302382921
MCH (α)	218533022	218533094	SIT (α)	99080974	99082115
MSL (α)	217977843	217976328	KVU (α)	310816538	310815351
HBA (α)	254294064	254294249	SJP (α)	294010880	294010306
SAL (α)	103486320	103488422	ELI (α)	85374135	85375723
AZL (α)	288958449	288959133	GOX (α)	58039721	58040203
SUA (ε)	307721946	307721047	GBE (α)	114327478	114328080
CJR (ε)	57238338	57237686	PBR (α)	304322092	304321587
HPY (ε)	15645104 (E1)	15645267 (Q1)	ACR (α)	148260952	148261658
WSU (ε)	34558490	34556712	BMC (α)	189024300	189024181
ANT (ε)	296271698	296273255	ECH (α)	88658087	88658242
NIS (ε)	152991382	152990548	MMR (α)	114569946	114569537
SKU (ε)	313683490	313682530	OTS (α)	148284334	148285057
SDL (ε)	268680812	268679320	PDE (α)	119386425	119383635
SUN (ε)	152991704	152992729	RSP (α)	77462542	77464373
NSA (ε)	319957716	319957052	MLO (α)	13470828	13470967
ABA (ad)	94967215	94967082	NSE (α)	88608680	88608755
ACA (ad)	225873235	225874464	NAR (α)	87200047	87198989
FNO (ht)	154249462	154249365	RRU (α)	83592936	83592027
KOL (ht)	239618082	239618313	RPR (α)	15604472	15604193
PMO (ht)	160902068	160903261	APH (α)	88606767	88607805
TAF (ht)	217077624	217076407	AZC (α)	158423823	158423403
TMA (ht)	15644618 (E1/Q1)	15644103 (X)	BQU (α)	49474279	49474272

* Species names are represented by three-letter KEGG-codes (see additional files 1 and 2), followed by abbreviated phyla (α: alphaproteobacteria, ε: epsilonproteobacteria, γ: gammaproteobacteria, ad: acidobacteria, ht: hyperthermophilic bacteria). The experimentally known specificities of GluRS1 and GluRS2 from three species (HPY, TMA and AFE) are highlighted where E1, E2, Q1 and Q2 stand for capability to glutamylate $tRNA^{Glu1}$, $tRNA^{Glu2}$, $tRNA^{Gln1}$ and $tRNA^{Gln2}$ respectively (X indicates that the GluRS is incapable of charging $tRNA^{Glu}$ or $tRNA^{Gln}$).
