# Supplementary material for: Evolutionary insights about bacterial GlxRS from whole genome analyses: is GluRS2 a chimera?
Source: BMC Evol Biol. 2014 Feb 12;14:26. doi: 10.1186/1471-2148-14-26 (PMC3927822; doi:10.1186/1471-2148-14-26)
Supplement: Additional file 6 — Sequence length distribution of bacterial GlnRS. [file 1471-2148-14-26-S6.pdf]

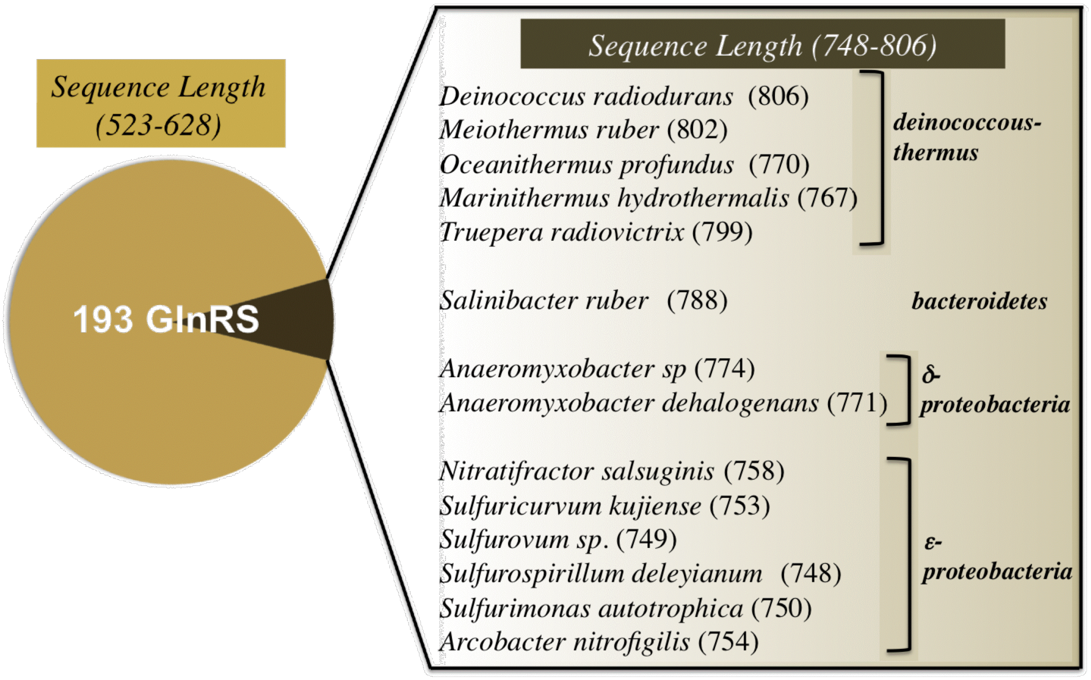

Sequence length distribution of bacterial GlnRS. GlnRS sequences with an appended Yqey domain (sequence length 748-806 amino acids) are highlighted.
