# Supplementary material for: Evolutionary insights about bacterial GlxRS from whole genome analyses: is GluRS2 a chimera?
Source: BMC Evol Biol. 2014 Feb 12;14:26. doi: 10.1186/1471-2148-14-26 (PMC3927822; doi:10.1186/1471-2148-14-26)
Supplement: Additional file 7 — Multiple-aligned tRNAGln sequences from GlnRS-containing bacteria. [file 1471-2148-14-26-S7.pdf]

```
              12345678901234567--890ab1234567890123-456-78901234567---8901234567890123456789012 3
OCA_10_al_D+  UGGGGCGUCGCCAAGU---GGU--AAGGCAGGGGAUU-UUG-AUUCCCCCAUG---CGGAGGUUCGAAUCCUCCCGCCCCAG
BJA_10_al_D+  UGGGGCGUAGCCAAGC---GGU--AAGGCAGCGGAUU-UUG-AUUCCGCCAUU---CGGAGGUUCGAUCCCUCCCGCCCCAG
NHA_10_al_D+  UGGGGCGUAGCCAAGC---GGU--AAGGCAGGGGAUU-UUG-AUUCCCCCAUG---CGGAGGUUCGAUCCCUCCCGCCCCAG
RPD_10_al_D+  UGGGGCGUAGCCAAGC---GGU--AAGGCAGGGGAUU-UUG-AUUCCCCCAUG---CGGAGGUUCGAUCCCUCCCGCCCCAG
OCA_20_al_D+  UGGGGAAUGGUGUAAC---GGU--AGCACAACAGACU-CUG-ACUCUGUUUG----UCUUGGUUCGAAUCCAGGUUCCCCAG
BJA_20_al_D+  UGGGGAAUGGUGUAAC---GGU--AGCACAACAGACU-CUG-ACUCUGUUUG----UCUUGGUUCGAAUCCAGGUUCCCCAG
NHA_20_al_D+  UGGGGAAUGGUGUAAC---GGU--AGCACAACAGACU-CUG-ACUCUGUUUG----UCUAGGUUCGAAUCCUAGUUCCCCAG
RPD_20_al_D+  UGGGGAAUGGUGUAAC---GGU--AGCACAACAGACU-CUG-ACUCUGUUUG----UCUAGGUUCGAAUCCUAGUUCCCCAG
APL_10_ga_D-  UGGGGUAUCGCCAAGC---GGU--AAGGCACCGGGUU-UUG-AUCUCGGCAUC---CCUAGGUUCGAAUCCUAGUACCCCAG
AHA_1A_ga_D-  UGGGGUAUCGCCAAGC---GGU--AAGGCAGCGGGUU-UUG-AUCUCGCCAUU---CCUAGGUUCGAAUCCUAGUACCCCAG
AHA_1B_ga_D-  UGGGGUAUCGCCAAGC---GGU--AAGGCAGCGGGUU-UUG-AUCUCGCCAUC---CCUAGGUUCGAAUCCUAGUACCCCAG
BCI_10_ga_D-  UGGGAUAUAGCCAAGU---GGU--AAGGCAGCGGGUU-UUG-AUCCCGCCAU----CCCAGGUUCAAAUCCUGGUAUCCCAG
BUC_10_ga_D-  UGGGAUAUAGCCAAGU---GGU--AAGGCACCGGUUU-UUG-AUACCGGCAU----CCCUGGUUCGAAUCCAGGUAUCCCAG
BFL_10_ga_D-  UGGGGUAUAGCCAAGC---GGU--AAGGCAGCGGGUU-UUG-AUCCCGCCACU---CCCAGGUUCGAAUCCUGGUACCCCAG
CPS_10_ga_D-  AGGGAUAUAGCCAAGC---GGU--AAGGCAGCGGGUU-UUG-AUCCCGUCAUU---CAGAGGUUCAAAUCCUCUUAUCCCUG
ECO_10_ga_D-  UGGGGUAUCGCCAAGC---GGU--AAGGCACCGGUUU-UUG-AUACCGGCAUU---CCCUGGUUCGAAUCCAGGUACCCCAG
HDU_10_ga_D-  UGGGGUAUCGCCAAGC---GGU--AAGGCACCGGGUU-UUG-AUCUCGGCAUC---CCUAGGUUCGAAUCCUAGUACCCCAG
ILO_10_ga_D-  UGGGGCAUAGCCAAGC---GGU--AAGGCAGCGGGUU-UUG-AUCCCGCGAUU---CCCAGGUUCGAUCCCUGGUGCCCCAG
MSU_10_ga_D-  UGGGGUAUCGCCAAGC---GGU--AAGGCACUGGGUU-UUG-AUCUCAGCAUU---CCUAGGUUCGAAUCCUAGUACCCCAG
PMU_10_ga_D-  UGGGGUAUCGCCAAGC---GGU--AAGGCACCGGGUU-UUG-AUCUCGGCAUU---CCUAGGUUCGAAUCCUAGUACCCCAG
PLU_10_ga_D-  UGGGGUAUCGCCAAGC---GGU--AAGGCACCAGGUU-UUG-AUCCUGGCAUA---CCCAGGUUCGAAUCCUGGUACCCCAG
PPR_1A_ga_D-  AGGGCUAUCGCCAAGC---GGU--AAGGCAGCGGCUU-UUG-AUGCCGCCAUC---CCCUGGUUCGAAUCCAGGUAGCCCUG
PPR_1B_ga_D-  AGGGCUAUCGCCAAGC---GGU--AAGGCAGCGGCUU-UUG-AUGCCGCCAUC---CCCUGGUUCGAAUCCAGGUGGCUCUG
PPR_1C_ga_D-  AGGGCUAUCGCCAAGC---GGU--AAGGCAGCGGCUU-UUG-AUGCCGCCAUC---CCCUGGUUCGAAUCCAGGUAGCUCUG
PAT_10_ga_D-  UGGGAUAUCGCCAAGU---GGU--AAGGCAACGGGUU-UUG-AUCCCGUCAUU---CGUUGGUUCGAAUCCAGCUAUCCCAG
PIN_10_ga_D-  UGGGAUAUCGCCAAGC---GGU--AAGGCACCGGGUU-UUG-AUCUCGGCAUU---CAGAGGUUCAAAUCCUCUUAUCCCAG
STT_10_ga_D-  UGGGGUAUCGCCAAGC---GGU--AAGGCACCGGUUU-UUG-AUACCGGCAUU---CCCUGGUUCGAAUCCAGGUACCCCAG
SDY_10_ga_D-  UGGGGUAUCGCCAAGC---GGU--AAGGCACCGGUUU-UUG-AUACCGGCAUU---CCCUGGUUCGAAUCCAGGUACCCCAG
SGL_1A_ga_D-  UGGGGUAUAGCCAAGC---GGU--AAGGCAGCGGGUU-UUG-AUCCCGCCACC---CCCAGGUUCGAAUCCUGGUACCCCAG
SGL_1B_ga_D-  UGGGAUAUAGCCAAGC---GGU--GAGGCAGCGGGUU-UUG-AUCCCGCCACC---CCCAGGUUCGAAUCCUGGUAUCCCAG
VPA_1A_ga_D-  AGGUCUAUCGCCAAGC---GGU--AAGGCAGCGGCUU-UUG-AUGCCGCCAUU---CCCUGGUUCGAAUCCAGGUAGACCUG
VPA_1B_ga_D-  AGGGCUAUCGCCAAGC---GGU--AAGGCAGCGGCUU-UUG-AUGCCGCCAUU---CCCUGGUUCGAAUCCAGGUAGCCCUG
XOP_10_ga_D-  AGGGGCGUCGCCAAGA---GGCCCAAGGCACCAGGUU-UUG-AUCCUGGCAUU---CGUAGGUUCGAAUCCUACCGCCCCUG
XFA_10_ga_D-  UGGGGCGUCGCCAAGA---GGACUAAGGCACCGGGUU-UUG-AUCCCGGCAUU---CGUAGGUUCGAAUCCUACCGCCCCUG
YPI_10_ga_D-  UGGGAUAUCGCCAAGC---GGU--AAGGCACCAGGUU-UUG-AUCCUGGCAUU---CCCAGGUUCGAAUCCUGGUAUCCCAG
WBR_10_ga_D-  UGGGGUAUAGCCAAGA---GGA--AAGGCAGCGGGUU-UUG-AUCCCGCGAU----CCCAGGUUCGAAUCCUGGUGCCCCAG
ENT_10_ga_D-  UGGGGUAUCGCCAAGC---GGU--AAGGCACCGGUUU-UUG-AUACCGGCAUU---CCCUGGUUCGAAUCCAGGUACCCCAG
ESA_1A_ga_D-  UGGGGUAUCGCCAAGC---GGU--AAGGCACCGGUUU-UUG-AUACCGGCAUU---CCCUGGUUCGAAUCCAGGUACCCCAG
ESA_1B_ga_D-  UGGGGUAUCGCCAAGC---GGU--AAGGCACCGGUUU-UUG-AUACCGGCAUC---CCCUGGUUCGAAUCCAGGUACCCCAG
CRO_1A_ga_D-  UGGGGUAUCGCCAAGC---GGU--AAGGCACCGGUUU-UUG-AUACCGGCAUU---CCCUGGUUCGAAUCCAGGUACCCCAG
CRO_1B_ga_D-  UGGGGUAUCGCCAAGC---GGU--AAGGCACCGGUUU-UUG-AUACCGGCAUC---CCCUGGUUCGAAUCCAGGUACCCCAG
PMR_1A_ga_D-  UGGGGUAUCGCCAAGC---GGU--AAGGCACCAGGUU-UUG-AUCCUGGCAUG---CCCUGGUUCGAAUCCAGGUACCCCAG
PMR_1B_ga_D-  UGGGGUAUCGCCAAGC---GGU--AAGGCACCAGGUU-UUG-AUCCUGGCAUU---CCCAGGUUCGAAUCCUGGUACCCCAG
PMR_1C_ga_D-  UGGGGUAUCGCCAAGC---GGU--AAGGCACCAGGUU-UUG-AUCCUGGCAUA---CCCUGGUUCGAAUCCAGGUACCCCAG
EIC_1A_ga_D-  UGGGGUAUCGCCAAGC---GGU--AAGGCAGCGGGUU-UUG-AUCCCGCCAUU---CCCAGGUUCGAAUCCUGGUACCCCAG
EIC_1B_ga_D-  UGGGGUAUCGCCAAGC---GGU--AAGGCAGCGGGUU-UUG-AUCCCGCCAUC---CCCAGGUUCGAAUCCUGGUACCCCAG
HDE_10_ga_D-  UGGGGUAUAGCCAAGU---GGU--AAGGCACCGGGUU-UUG-AUCCCGGCAUU---CCCAGGUUCGAAUCCUGGUACCCCAG
XBO_10_ga_D-  UGGGGUAUCGCCAAGC---GGU--AAGGCACCAGGUU-UUG-AUCCUGGCAUG---CCCUGGUUCGAAUCCAGGUACCCCAG
RIP_10_ga_D-  UGGGGUAUAGCCAAGC---GGA--AAGGCACCAGGUU-UUG-AUCCUGGCAU----CCCUGGUUCGAAUCCAGGUACCCCAG
RAH_1A_ga_D-  UGGGGUAUCGCCAAGC---GGU--AAGGCACUGGUUU-UUG-AUACCAGCAUU---CCCAGGUUCGAAUCCUGGUACCCCAG
RAH_1B_ga_D-  UGGGGUAUCGCCAAGC---GGU--AAGGCACUGGUUU-UUG-AUACCAGCAUA---CCCUGGUUCGAAUCCAGGUACCCCAG
MEN_10_ga_D-  UGGGAUAUAGCCAAGC---GGU--AAGGCAGCGGGUU-UUG-AUCCCGCCACA---CCCAGGUUCGAAUCCUGGUAUCCCAG
HSO_10_ga_D-  UGGGGUAUCGCCAAGC---GGU--AAGGCACCGGGUU-UUG-AUCUCGGCAUU---CCUAGGUUCGAAUCCUAGUACCCCAG
GAN_10_ga_D-  UGGGGUAUCGCCAAGC---GGU--AAGGCACCGGGUU-UUG-AUCUCGGCAUC---CCUAGGUUCGAAUCCUAGUACCCCAG
SML_10_ga_D-  UGGGGCGUCGCCAAGA---GGCCCAAGGCACCAGGUU-UUG-AUCCUGGCAUU---CGUAGGUUCGAAUCCUACCGCCCCUG
PSU_10_ga_D-  AGGGGCGUCGCCAAGU---GGUCCAAGGCACCGGGUU-UUG-AUCCCGGCAUC---CGCAGGUUCGAAUCCUGCCGCCCCUG
VAN_1A_ga_D-  AGGGCUAUCGCCAAGC---GGU--AAGGCAGCGGCUU-UUG-AUGCCGCCAUC---CCCUGGUUCGAAUCCAGGUAGCCCUG
VAN_1B_ga_D-  AGGGCUAUCGCCAAGC---GGU--AAGGCAGCGGCUU-UUG-AUGCCGCCAUU---CCCUGGUUCGAAUCCAGGUAGCCCUG
AMK_10_ga_D-  UGGGGUAUAGCCAAGUU--GGU--AAGGCAGCGGGUU-UUG-AUCCCGCCAUU---CGUAGGUUCGAGUCCUGCUACCCCAG
FBL_10_ga_D-  UGGGCUAUCGCCAAGC---GGU--AAGGCAGCGGGUU-UUG-AUCCCGCCAUU---CCCAGGUUCGAAUCCUGGUAGCCCAG
KKO_10_ga_D-  AGGGGUAUCGCCAAGC---GGU--AAGGCAGCGGCUU-UUG-AUGCCGCCAUU---CGUUGGUUCAAAUCCAGCUACCCCUG
SPE_10_ga_D+  UGGGGUAUCGCCAAGC---GGU--AAGGCACUGGUUU-UUG-AUACCAGCAUU---CCCUGGUUCGAAUCCAGGUACCCCAG
LPN_10_ga_D+  AGGGGUGUCGCCAAGC---GGU--AAGGCACAGGGUU-UUG-AUCCCUGCAUA---CCUAGGUUCGAAUCCUAGCACCCCUG
TCX_10_ga_D+  UGGGCUAUAGCCAAGC---GGU--AAGGCAACUGGUU-UUG-AUCCAGUCAUG---CGCAGGUUCGAAUCCUGCUAGCCCAG
PAE_10_ga_D+  AGGGGCGUCGCCAAGC---GGU--AAGGCAGCAGGUU-UUG-AUCCUGCCAUG---CGUUGGUUCGAAUCCAGCCGCCCCUG
SDE_10_ga_D+  UGGGGUGUAGCCAAGC---GGU--AAGGCAGCGGGUU-UUG-AUCCCGUCAUG---CGAAGGUUCGAAUCCUUCCACCCCAG
PAR_10_ga_D+  AGGGGUAUAGCCAAGUU--GGU--AAGGCAUCAGGUU-UUG-AUCCUGACAUC---CGUUGGUUCGAGUCCAGCUACCCCUA
HCH_1B_ga_D+  UGGGGUGUAGCCAAGC---GGU--AAGGCAGCGGGUU-UUG-AUCCCGUCAUG---CGUAGGUUCGAAUCCUACCACCCCAG
KPN_10_ga_D+  UGGGGUAUCGCCAAGC---GGU--AAGGCACCGGUUU-UUG-AUACCGGCAUU---CCCUGGUUCGAAUCCAGGUACCCCAG
DDA_10_ga_D+  UGGGGUAUCGCCAAGC---GGU--AAGGCAGCGGGUU-UUG-AUCCCGCCAUC---CCCAGGUUCGAAUCCUGGUACCCCAG
PAM_10_ga_D+  UGGGAUAUCGCCAAGC---GGU--AAGGCACCGGUUU-UUG-AUACCGGCAUU---CCCUGGUUCGAAUCCAGGUAUCCCAG
GAG_10_ga_D+  UGGGAUAUCGCCAAGU---GGU--AAGGCAACGGGUU-UUG-AUCCCGUCAUU---CGUUGGUUCGAAUCCAGCUAUCCCAG
MMT_10_ga_D+  UGGGCUGUCGCCAAGC---GGU--AAGGCACGGGGUU-UUG-AUCUCCGCAUA---CCUAGGUUCGAAUCCUAGCAGCCCAG
MAH_10_ga_D+  UGGGCCGUCGCCAAGC---GGU--AAGGCACGGGGUU-UUG-AUCCCCGCAUA---CCCAGGUUCGAAUCCUGGCGGCCCAG
TCY_10_ga_D+  UGGGCUGUAGCCAAGC---GGU--AAGGCAGCGGGUU-UUG-AUCCCGUCAUG---CGAAGGUUCGAAUCCUUCCAGCCCAG
HNA_10_ga_D+  UGGGCCGUCGCCAAGUU--GGUU-AAGGCACUGGGUU-UUG-AUCCCAGCAUU---CCAAGGUUCGAAUCCUUGCGGCCCAG
TAU_10_ga_D+  UGGGGUAUCGCCAAGC---GGU--AAGGCAACGGGUU-UUG-AUCCCGUCAUA---CCCAGGUUCGAAUCCUGGUACCCCAG
DNO_10_ga_D+  UGGGGUGUCGCCAAGU---GGU--AAGGCAUCGGGUU-UUG-AUCCCGACAUG---CGUAGGUUCGAAUCCUGCCACCCCAG
```

```
                  12345678901234567--890ab1234567890123-456-78901234567---890123456789012345678901234567890123
RMA_10_ga_D+   UGGGCUGUCGCCAAGC---GGU--AAGGCAACGGGUU-UUG-AUCCCGUCAUU---CGCAGGUUCAAAUCCUGCCAGCCCAG
VOK_10_ga_D+   UGGGCUGUCGCCAAGC---GGU--AAGGCAACGGGUU-UUG-AUCCCGUCAUA---CGCAGGUUCAAAUCCUGCCAGCCCAG
AVN_10_ga_D+   AGGGGCGUCGCCAAGC---GGU--AAGGCAGCAGGUU-UUG-AUCCUGCCAUG---CGUUGGUUCGAAUCCAGCCGCCCCUG
ACI_1A_ga_D+   AGGGGCGUCGCCAAGU---GGU--AAGGCACCGGGUU-UUG-AUCUCGGCAUC---CGUUGGUUCGAAUCCAGCCGCCCCUG
ACI_1B_ga_D+   AGGGGCGUCGCCAAGU---GGU--AAGGCAGCGGGUU-UUG-AUCUCGCCAUC---CGUUGGUUCGAAUCCAGCCGCCCCUG
MCT_10_ga_D+   AGGGGUAUCGCCAAGUU--GGU--AAGGCAUCAGGUU-UUG-AUCCUGACAUU---CGUUGGUUCGAGUCCAGCUACCUCUG
MAQ_10_ga_D+   UGGGGUGUCGCCAAGU---GGU--AAGGCAACGGGUU-UUG-AUCCCGUCAUG---CGCAGGUUCGAAUCCUGCCACCCCAG
CSA_10_ga_D+   UGGGGUAUAGCCAAGU---GGU--AAGGCACCGGUUU-UUG-GUAUCGGCAUU---CGCAGGUUCGAGUCCUGCUACCCCAG
HEL_1A_ga_D+   UGGGGUAUAGCCAAGU---GGU--AAGGCAGCGGUUU-UUG-GUAUCGCCAUG---CGCAGGUUCGAAUCCUGCUACCCCAG
HEL_1B_ga_D+   UGGGGUAUCGCCAAGU---GGU--AAGGCACUGGUUU-UUG-GUAUCAGCAUG---CGCAGGUUCGAAUCCUGCUACCCCAG
ABO_10_ga_D+   UGGGGUAUAGCCAAGUU--GGU--AAGGCAGCGGGUU-UUG-AUCCCGUUAUU---CCCAGGUUCGAGUCCUGGUACCCCAG
MMW_10_ga_D+   UGGGGUAUAGCCAAGC---GGU--AAGGCAACGGGUU-UUG-AUCCCGUCAUG---CGUAGGUUCGAAUCCUUCUACCCCAG
CJA_10_ga_D+   AGGGGCGUCGCCAAGU---GGU--AAGGCAGCGGGUU-UUG-AUCCCGCCAUU---CGUAGGUUCGAGUCCUUCCGCCCCUG
ALV_10_ga_D+   UGGGGUGUCGCCAAGC---GGU--AAGGCACCGGGUU-UUG-AUCCCGGCAUU---CCCAGGUUCGAAUCCUGGCACCCCAG
TTU_10_ga_D+   UGGGGUGUCGCCAAGC---GGU--AAGGCAACGGGUU-UUG-AUCCCGUCAUG---CGGAGGUUCGAAUCCUCCCACCCCAG
GPB_10_ga_D+   UGGGCUGUCGCCAAGC---GGU--AAGGCACUGGAUU-UUG-AUUCCAGCAUU---CCCAGGUUCGAAUCCUGGCAGCCCAG
FTT_10_ga_D+   UGGGCUAUCGCCAAGC---GGU--AAGGCAACGGGUU-UUG-AUCCCGUCAUU---CCCAGGUUCGAAUCCUGGUAGCCCAG
ECO_20_ga_D-   UGGGGUAUCGCCAAGC---GGU--AAGGCACCGGAUU-CUG-AUUCCGGCAUU---CCGAGGUUCGAAUCCUCGUACCCCAG
BCI_20_ga_D-   UGGGGUAUAGCUAAGU---GGU--AAGGCACCGGAUU-CUG-GUUCCGGCAUU---CCUAGGUUCGAAUCCUAGUACCCCAG
PLU_20_ga_D-   UGGGGUAUCGCCAAGC---GGU--AAGGCACCGGAUU-CUG-AUUCCGGCAUU---CCCAGGUUCGAAUCCUGGUACCCCAG
SDY_20_ga_D-   UGGGGUAUCGCCAAGC---GGU--AAGGCACCGGAUU-CUG-AUUCCGGCAUU---CCGAGGUUCGAAUCCUCGUACCCCAG
SGL_20_ga_D-   UGGGGUAUCGCCAAGU---GGU--AAGGCACCGGAUU-CUG-AUUCCGGCACC---CCCAGGUUCGAAUCCUGGUACCCCAG
XOP_20_ga_D-   UGCCCCGUCGCCAAGC---GGU--AAGGCACCUGACU-CUG-ACUCAGGCAU----CGGUGGUUCGAAUCCAUCCGGGGCAG
XFA_20_ga_D-   UGCCCCGUAGCCAAGU---GGU--AAGGCAUCUGACU-CUG-ACUCAGACAUU---CGUUGGUUCGAAUCCAUCCGGGGCAG
YPI_20_ga_D-   UGGGGUAUCGCCAAGC---GGU--AAGGCACUGGAUU-CUG-AUUCCAGCAUU---CCGAGGUUCGAAUCCUCGUACCCCAG
ENT_2A_ga_D-   UGGGGUGUCGCCAAGC---GGU--AAGGCACUGGUUU-CUG-AUACCAGCAUU---CCGGGGUUCGAAUCCCUGCACCCCAG
ENT_2B_ga_D-   UGGGGUAUCGCCAAGC---GGU--AAGGCUCUGGUUU-CUG-AUACCAGCAUU---CCGAGGUUCGAAUCCUCGUACCCCAG
ESA_20_ga_D-   UGGGGUGUCGCCAAGC---GGU--AAGGCAGCGGAUU-CUG-AUUCCGCCAUU---CCGGGGUUCGAAUCCCUGCACCCCAG
CRO_20_ga_D-   UGGGGUGUCGCCAAGC---GGU--AAGGCAGCGGAUU-CUG-AUUCCGCCAUU---CCGGGGUUCGAAUCCCUGCACCCCAG
EIC_20_ga_D-   UGGGGUAUCGCCAAGC---GGU--AAGGCAGCGGAUU-CUG-AUUCCGCCAUC---CCGAGGUUCGAAUCCUCGUACCCCAG
HDE_20_ga_D-   UGGGGUAUAGCCAAGC---GGU--AAGGCAUCGGAUU-CUG-AUUCCGACAUU---CCCAGGUUCAAAUCCUGGUACCCCAG
XBO_20_ga_D-   UGGGGUAUCGCCAAGC---GGU--AAGGCACCGGAUU-CUG-AUUCCGGCAU----CCCAGGUUCGAAUCCUGGUACCCCAG
RAH_20_ga_D-   UGGGGUGUCGCCAAGC---GGU--AAGGCUCUGGUUU-CUG-AUACCAGCAUA---CCCAGGUUCGAAUCCUGGCACCCCAG
MEN_20_ga_D-   UGGGGUAUAGCCAAGC---GGU--AAGGCACCGGAUU-CUG-AUUCCGGCAUC---CCCAGGUUCGAAUCCUGGUACCCCAG
GAN_20_ga_D-   UGGGGUAUCGCCAAGC---GGU--AAGGCACCGGAUU-CUG-AUUCCGGCAUU---CCUAGGUUCGAAUCCUAGUACCCCAG
SML_20_ga_D-   UGCCCCGUCGCCAAGC---GGU--AAGGCACCUGACU-CUG-ACUCAGGCAUU---CGGUGGUUCGAAUCCAUCCGGGGCAG
PSU_20_ga_D-   UGCCCCGUCGCCAAGC---GGU--AAGGCACCUGACU-CUG-ACUCAGGCAUU---CGGAGGUUCGAAUCCUUCCGGGGCUG
SPE_20_ga_D+   UGGGGUGUCGCCAAGC---GGU--AAGGCUCUGGUUU-CUG-AUACCAGCAUA---CCCAGGUUCGAAUCCUGGCACCCCAG
KPN_20_ga_D+   UGGGGUAUCGCCAAGC---GGU--AAGGCACCGGAUU-CUG-AUUCCGGCAUU---CCGAGGUUCGAAUCCUCGUACCCCAG
DDA_20_ga_D+   UGGGGUAUCGCCAAGC---GGU--AAGGCAGCGGAUU-CUG-AUUCCGCCAUC---CCGAGGUUCGAAUCCUCGUACCCCAG
TAU_20_ga_D+   UGGGGUGUCGCCAAGC---GGU--AAGGCAGUGGAUU-CUG-AUUCCACCAUA---CCCAGGUUCGAAUCCUGGCACCCCAG
CSA_20_ga_D+   UGGGGUAUAGCCAAGU---GGUU-AAGGCACCGGUUU-CUG-GUACCGGCAUU---CCCAGGUUCAAAUCCUGGUACCCCAG
ALV_20_ga_D+   UGGGGAAUCGUCUAAC---GGC--AGGACAGCGGACU-CUG-ACUCCGUCAA----UCUAGGUUCGAAUCCUAGUUCCCCAG
PAM_20_ga_D+   UGGGGUGUCGCCAAGC---GGU--AAGGCACUGGUUU-CUG-AUACCAGCAUU---CCGGGGUUCGAAUCCCUGCACCCCAG
BBR_10_be_D+   UGGGGAGUCGCCAAGCU--GGUU-AAGGCACCGGAUU-UUG-AUUCCGGCAUG---CGAAGGUUCGAAUCCUUCCUCCCCAG
CVI_1A_be_D+   UGGGGAGUCGCCAAGU---GGU--AAGGCACCGGAUU-UUG-AUUCCGGCAUU---CGUAGGUUCGAUCCCUACCUCCCCAG
CVI_1B_be_D+   UGGCGGUUAGCUCAGUU--GGU--AGAGCAUCGGAUU-UUG-AUUCCGAGGGU---CACAGGUUCGAGCCCUGUACCGCCUG
DAR_10_be_D+   UGGGGAGUCGCCAAGUU--GGUC-AAGGCACCGGAUU-UUG-AUUCCGGCAUU---CGAAGGUUCGAAUCCUUCUUCCCCAG
MFA_10_be_D+   UGGGGAGUCGCCAAGCU--GGUU-AAGGCACCGGAUU-UUG-AUUCCGGCAUG---CGAAGGUUCGAAUCCUUCCUCCCCAG
NMU_10_be_D+   UGGGGAGUCGCCAAGU---GGU--AAGGCACCGGAUU-UUG-AUUCCGGCAUU---CGUAGGUUCGAUCCCUACCUCCCCAG
RSO_10_be_D+   AGGGGAGUCGCCAAGUU--GGUU-AAGGCACCGGAUU-UUG-AUUCCGGCAUG---CGAGGGUUCGAGUCCUUCCUCCCCUG
RFR_10_be_D+   AGGGGAGUCGCCAAGUU--GGUU-AAGGCACUGGAUU-UUG-AUUCCAGCAUG---CGAAGGUUCGAAUCCUUCUUCCCCUG
TBD_10_be_D+   UGGGGAGUCGCCAAGU---GGU--AAGGCAUCGGAUU-UUG-AUUCCGACAUU---CGUAGGUUCGAUCCCUACCUCCCCAG
NMC_10_be_D+   UGGGGAGUCGUCAAGC---GGUU-AAGACACUGGAUU-UUG-AUUCCAGCAUG---CGAAGGUUCGAAUCCUUCCUCCCCAG
LHK_10_be_D+   UGGGGAGUCGCCAAGU---GGU--AAGGCACCGGAUU-UUG-AUUCCGGCAUU---CGUAGGUUCGAUCCCUACCUCCCCAG
PSE_10_be_D+   UGGGGAGUCGCCAAGU---GGU--AAGGCACCGGAUU-UUG-AUUCCGGCAUU---CGUAGGUUCGAUCCCUACCUCCCCAG
BMA_10_be_D+   AGGGGAGUCGCCAAGUU--GGUU-AAGGCACCGGAUU-UUG-AUUCCGGCAUG---CGAGGGUUCGAGUCCUUCCUCCCCUG
PNU_10_be_D+   AGGGGAAUCGCCAAGCU--GGUU-AAGGCACUGGAUU-UUG-AUUCCAGCAUG---CGAAGGUUCGAAUCCUUCUUCCCCUG
AXY_10_be_D+   UGGGGAGUCGCCAAGCU--GGUU-AAGGCACCGGAUU-UUG-AUUCCGGCAUG---CGAAGGUUCGAAUCCUUCCUCCCCAG
TEQ_10_be_D+   UGGGGCGUCGCCAAGCU--GGUU-AAGGCACCGGAUU-UUG-AUUCCGGCAUG---CGAAGGUUCGAAUCCUUCCGCCCCAG
PUT_10_be_D+   UGGGGAAUCGCCAAGCU--GGUU-AAGGCACUGGAUU-UUG-AUUCCAGCAUG---CGAAGGUUCGAAUCCUUCUUCCCCAG
POL_10_be_D+   AGGGGUGUCGCCAAGCU--GGUU-AAGGCACCGGAUU-UUG-AUUCCGGCAUU---CGCAGGUUCGAAUCCUUCCACCCCUG
AAV_10_be_D+   AGGGGAGUCGCCAAGCU--GGUU-AAGGCACCGGAUU-UUG-AUUCCGGCAUG---CGAAGGUUCGAAUCCUUCUUCCCCUG
VEI_10_be_D+   AGGGGAGUCGCCAAGCU--GGUU-AAGGCACCGGAUU-UUG-AUUCCGGCAUG---CGAAGGUUCGAAUCCUUCUUCCCCUG
DAC_10_be_D+   AGGGGAGUCGCCAAGUU--GGUA-AAGGCACCGGAUU-UUG-AUUCCGGCAUG---CGAGGGUUCGAGUCCUUCCUCCCCUG
VAP_1A_be_D+   AGGGGAGUCGCCAAGCU--GGUU-AAGGCACUGGAUU-UUG-AUUCCAGCAUG---CAAAGGUUCGAAUCCUUUCUCCCCUG
VAP_1B_be_D+   AGACCGUUCGCCAAGCU--GGACUAAGGCACCUGACU-UUG-ACUCAGGCAAU---CGCACGUUCGAAUCGUGCACGGUCUG
CTT_1A_be_D+   AGGGGAGUCGCCAAGUU--GGUU-AAGGCACCGGAUU-UUG-AUUCCGGCAUG---CGAAGGUUCGAAUCCUUCUUCCCCUG
CTT_1B_be_D+   AGGGGAGUCGCCAAGCU--GGUU-AAGGCACUGGAUU-UUG-AUUCCAGCAUG---CGAAGGUUCGAAUCCUUCCUCCCCUG
ADN_10_be_D+   AGGGGAGUCGCCAAGUU--GGUU-AAGGCACCGGAUU-UUG-AUUCCGGCAUG---CGAGGGUUCGAGUCCUUCCUCCCCUG
RTA_10_be_D+   AGGGGAGUCGCCAAGUU--GGUU-AAGGCACCGGAUU-UUG-AUUCCGGCAUG---CGAGGGUUCGAGUCCUUCCUCCCCUG
MPT_10_be_D+   AGGGGAGUCGCCAAGUU--GGUU-AAGGCAUCGGAUU-UUG-AUUCCGACAUG---CGAAGGUUCGAAUCCUUCUUCCCCUG
HAR_10_be_D+   AGGGGAAUCGCCAAGUU--GGUU-AAGGCACUGGAUU-UUG-AUUCCAGCAUU---CGAAGGUUCGAGUCCUUCUUCCCCUG
MMS_10_be_D+   AGGGGAAUCGCCAAGUU--GGUU-AAGGCACUGGAUU-UUG-AUUCCAGCAUU---CGAAGGUUCGAAUCCUUCUUCCCCUG
HSE_10_be_D+   AGGGGAAUCGCCAAGCU--GGUU-AAGGCACCGGAUU-UUG-AUUCCGGCAUU---CCAAGGUUCGAAUCCUUGUUCCCCUG
ZIN_10_be_D+   AGGGGAAUAGCCAAGUU--GGUUUAAGGCACCGGAUU-UUG-AUUCCGGCAU----CCAGGGUUCAAAUCCUUGUUCCCCUG
CFU_10_be_D+   AGGGGAAUCGCCAAGCU--GGUU-AAGGCACUGGAUU-UUG-AUUCCAGCAUU---CGAAGGUUCGAAUCCUUCUUCCCCUG
LCH_10_be_D+   AGGGGAGUCGCCAAGUU--GGUC-AAGGCAUCGGAUU-UUG-AUUCCGACAUG---CGAGGGUUCGAGUCCUUCCUCCCCUG
```

```
                  12345678901234567--890ab1234567890123-456-78901234567---890123456789012345678901234567890123
TIN_10_be_D+  UGGGGAGUCGCCAAGUU--GGUU-AAGGCACCGGAUU-UUG-AUUCCGGCAUU---CGAGGGUUCGAGUCCUUCCUCCCCAG
NEU_10_be_D+  UGGGGAGUCGCCAAGU---GGU--AAGGCACCGGAUU-UUG-AUUCCGGCAUU---CGUAGGUUCGAUCCCUACCUCCCCAG
EBA_10_be_D+  UGGGGAGUCGCCAAGUU--GGUC-AAGGCACCGGAUU-UUG-AUUCCGGCAUU---CGAAGGUUCGAAUCCUUCUUCCCCAG
AZO_10_be_D+  UGGGGAGUCGCCAAGUC--GGUU-AAGGCACCGGAUU-UUG-AUUCCGGCAUU---CGAGGGUUCGAAUCCUUCCUCCCCAG
TMZ_10_be_D+  UGGGGAGUCGCCAAGUC--GGUU-AAGGCACCGGAUU-UUG-AUUCCGGCAUU---CGAGGGUUCGAAUCCUUCCUCCCCAG
MMB_10_be_D+  UGGGGAGUCGCCAAGCU--GGUU-AAGGCACCGGAUU-UUG-AUUCCGGCAUG---CGAAGGUUCGAAUCCUUCCUCCUCAG
MEI_10_be_D+  UGGGGAGUCGCCAAGCU--GGUU-AAGGCACCGGAUU-UUG-AUUCCGGCAUG---CGAAGGUUCGAAUCCUUCUUCCCCAG
APP_10_be_D+  UGGGGAGUCGCCAAGUU--GGUU-AAGGCACCGGAUU-UUG-AUUCCGGCAUU---CGAAGGUUCGAAUCCUUCUUCCCCAG
SLT_10_be_D+  UGGGGAGUCGCCAAGU---GGU--AAGGCAUCGGAUU-UUG-AUUCCGACAUU---CGUAGGUUCGAUCCCUACCUCCCCAG
GCA_10_be_D+  UGGGGAGUCGCCAAGU---GGU--AAGGCAACGGGUU-UUG-AUCCCGUCAUU---CGUAGGUUCGAUCCCUACCUCCUCAG
VPE_10_be_D+  AGGGGAGUCGCCAAGCU--GGUU-AAGGCACUGGAUU-UUG-AUUCCAGCAUG---CAAAGGUUCGAAUCCUUUCUCCCCUG
RME_10_be_D+  AGGGGAGUCGCCAAGUU--GGUC-AAGGCACCGGAUU-UUG-AUUCCGGCAUU---CGAAGGUUCGAAUCCUUCUUCCCCUG
LIP_10_de_D+  UGGGGUGUCGCCAAGUU--GGU--AAGGCAACGGGUU-UUG-GUCCCGUCAUU---CGAGGGUUCGAGUCCUUCCGCCCCAG
AFW_10_de_D+  UGAGGCGUCGUCCAAC---GGC--AGGACUGCGGACU-UUG-GAUCCGCGUA----UGAAGGUUCGAAUCCUUCCGCCUCAG
DVU_10_de_D+  UGGGGUGUCGCCAAGUU--GGU--AAGGCAACGGGUU-UUG-GUCCCGUCAUU---CGAGGGUUCGAGUCCUUCCGCCCCAG
MXA_10_de_D+  UGGGGCGUCGUCUAAU---GGC--AGGACAUCAGACU-UUG-ACUCUGAGUA----UCAAGGUUCGAAUCCUUGCGCCCCAG
PCA_10_de_D+  UGGGGCGUCGCCAAGC---GGU--AAGGCACCGGAUU-UUG-AUUCCGGCAUU---CGUAGGUUCGAAUCCUGCCGCCCCAG
ADE_10_de_D+  UGAGGCGUCGUCUAAU---GGC--AGGACCGCGGACU-UUG-GAUCCGCUCA----UGAAGGUUCGAAUCCUUCCGCCUCAG
GLO_10_de_D+  AGGGGUGUCGCCAAGC---GGU--AAGGCACCGGAUU-UUG-AUUCCGGCAUU---CCUAGGUUCGAAUCCUGGCACCCCUG
DAL_1A_de_D+  AGGGGCGUCGUCAAGC---GGU--AAGACACAUGGUU-UUG-GUCCAUGCAUU---CGGGGGUUCGAAUCCUCCCGCCCCUG
DAL_1B_de_D+  AGGGGUGUAGUUUAAUU--GGC--AAGACAUUGGGCC-UUG-GACCCAAUAC----UGCUGGUUCGAAUCCAGCCGCCCCUG
DOL_10_de_D+  UGGGGCGUCGUCAAGC---GGUA-AAGACACAGGAUU-UUG-AUUCCUGCAUU---CGGAGGUUCGAAUCCUCCCGCCCCAG
DPR_10_de_D+  UGGGGCGUCGUCAAGC---GGU--AAGACACAAGGUU-UUG-AUCCUUGCAUU---CGGGGGUUCGAAUCCUCCCGCCCCAG
DAK_10_de_D+  UGGGGCGUCGUCAAGC---GGU--AAGACACAAGACU-UUG-ACUCUUGCAUU---CGCAGGUUCGAAUCCUGCCGCCCCAG
DPS_10_de_D+  UGGGGCGUCGCCAAGC---GGU--AAGGCACCAGGUU-UUG-AUCCUGGCAUG---CGUAGGUUCGAAUCCUGCCGCCCCAG
DRT_10_de_D+  UGGGGUGUAGCCAAGU---GGU--AAGGCAACGGGUU-UUG-GUCCCGUCAUG---CGGGGGUUCAAAUCCUCCCACCCCAG
BBA_10_de_D+  UGGGGUAUCGUCAAGUU--GGU--AAGACAACAGAUU-UUG-AUUCUGUCAUU---CGCAGGUUCGAGUCCUGCUACCCCAU
DAT_10_de_D+  UGGGGCGUCGUCAAGU---GGU--AAGACACAGGAUU-UUG-AUUCCUGCAUU---CGGAGGUUCGAACCCUCCCGCCCCAG
DAO_10_de_D+  UGGGGGAUCGUCCAAU---GGC--AGGACAGCGGCCU-UUG-GAGUCGCCGA----UAGAGGUUCGAAUCCUCUUCCCCCAG
SAT_10_de_D+  UGGGGCGUCGUCAAGC---GGU--AAGACACAAGAUU-UUG-GUUCUUGCAUG---CGGAGGUUCGAAUCCUCCCGCCCCAG
SUR_10_de_D+  UGGGGCGUCGUCUAAU---GGC--AGGACGUCAGACU-UUG-AAUCUGAUUA----UCAAGGUUCGAAUCCUUGCGCCCCAG
HOH_10_de_D+  UCGGGGAUCGUCUAAA---GGC--AGGACAAUGGUUU-UUG-GUACCAUUUA----UGAAGGUUCGAAUCCUUCUCCCCGAA
SFU_10_de_D+  UGGGGCGUCGCCAAGC---GGU--AAGGCACGGGUCU-UUG-GAACCCGCACC---CGGAGGUUCGAAUCCUCCCGCCCCAG
DBR_10_de_D+  UGGGGUGUCGUCAAGC---GGU--AAGACACGGGUCU-UUG-GAACCCGCAUU---CGUAGGUUCGAAUCCUACCACCCCAG
HMR_10_de_D+  UGGGGCGUAGCCAAGU---GGU--AAGGCAGAGGACU-UUG-GAUCCUCGAU----CGCUGGUUCGAAUCCAGCCGCCCCAG
DBA_10_de_D+  UGGGGCGUCGCCAAGCU--GGC--AAGGCAACGGAUU-UUG-GUUCCGUCAUC---CGGAGGUUCGAAUCCUCCCGCCCCAG
LIP_20_de_D+  UGGGCUGUCGUUCAAU---GGC--AGGACAGCGGAUU-CUG-ACUCCGUUAA----UCAAGGUUCAAGUCCUUGCAGCCCAG
AFW_20_de_D+  UGGGGGAUCGUCUAAC---GGC--AGGACGCCAGUCU-CUG-GAUCUGGCUA----UCUAGGUUCGAAUCCUAGUCCCCCAG
DVU_20_de_D+  UGGGCUGUCGUUCAAUU--GGC--AGGACGACGGAUU-CUG-GCUCCGUUAA----UCUAGGUUCGAGUCCUAGCAGCCCAG
MXA_20_de_D+  UGGGGAAUCGUCUAAC---GGC--AGGACAGCAGACU-CUG-ACUCUGCUUA----UCUAGGUUCGAAUCCUAGUUCCCCAG
PCA_20_de_D+  UGGGAGAUCGUCUAGC---GGC--AGGACUACGGACU-CUG-ACUCCGUCAA----CCAAGGUUCGAAUCCUUGUCUCCCAG
ADE_20_de_D+  UGGGGGAUCGUCUAAC---GGC--AGGACGCCAGACU-CUG-GAUCUGGCUA----UCUAGGUUCGAAUCCUAGUCCCCCAG
DAL_20_de_D+  UGAGGGAUCGUCUAGU---GGC--AGGACGACGGGUU-CUG-GCCCCGUUAA----CCAAGGUUCGAAUCCUUGUCCCUCAG
DOL_20_de_D+  UGAGGGAUCGUCUAAU---GGC--AGGACGACGGGUU-CUG-GCCCCGUUAA----UCAAGGUUCGAAUCCUUGUCCCUCAG
DPR_20_de_D+  UGGGGGAUGGUCUAAC---GGC--AAGACAGCGGACU-CUG-ACUCCGCUUA----UCGGGGUUCGAAUCCCUGUCCCCCAG
DAK_20_de_D+  UGGGGGAUCGUCUAAU---GGU--AGGACUGCAGACU-CUG-ACUCUGCCUG----UCGGGGUUCGAAUCCCUGUCCCCCAG
DRT_20_de_D+  UGGGGGAUCGUCUAGG---GGC--AGGACAACGGAUU-CUG-GUUCCGUCAG----CCUAGGUUCGAAUCCUAGUCCCCCAG
DAT_20_de_D+  UGGGUGAUCGUCCAAU---GGC--AGGACUACGGACU-CUG-ACUCCGUCAA----UUAAGGUUCGAAUCCUUAUCACCCAG
DAO_20_de_D+  UGGGGGAUCGUCUAGC---GGC--AGGACAGCAGACU-CUG-GAUCUGCCUA----CCGAGGUUCGAAUCCUCGUCCCCCAG
SAT_20_de_D+  UGGGGAAUCGUUCAAU---GGU--AGGACAACGGACU-CUG-ACUCCGUGAA----UAUAGGUUCGAAUCCUGUUUCCCCAG
SUR_20_de_D+  UGGGGAAUCGUCUAAC---GGC--AGGACAGCAGACU-CUG-ACUCUGCUUA----UCUAGGUUCGAAUCCUAGUUCCCCAG
HOH_20_de_D+  UCGGGAAUCGUCUAAU---GGU--AGGACUGCAGACU-CUG-GAUCUGCCUA----UCUAGGUUCGAGUCCUAGUUCCCGAA
SFU_20_de_D+  UGGGGGAUCGUCCAGC---GGC--AGGACUGCAGACU-CUG-GAUCUGCCUA----CCUAGGUUCGAAUCCUAGUCCCCCAG
DBR_20_de_D+  UGGCGGAUCGUCUAAA---GGC--AGGACGACGGGUU-CUG-GCCCCGUUAA----UCUAGGUUCGAAUCCUAGUCCGCCAG
HMR_20_de_D+  UGGGAUGUCGUCUAAC---GGU--AGGACAGCGGACU-CUG-GAUCCGCGAG----UCGGGGUUCGAAUCCCUGCAUCCCAG
DBA_20_de_D+  UGGGUGAUCGUUCAAC---GGU--AGGACAGCGGACU-CUG-ACUCCGUCAA----UCUUAGUUCGAAUCUAAGUCACCCAG
SUS_10_ad_D+  UCCCCGGUCGUCUAAU---GGU--AGGACAGCGGCCU-UUG-GAGCCGUGAA----UCGUGGUUCGAAUCCAUGCCGGGGAG
SUS_20_ad_D+  UGGGACGUCGUCCAAC---GGU--AGGACUGCAGCCU-CUG-GAGCUGCGUA----UCGGGGUUCGAAUCCCUGCGUCCCAG
ATM_10_NS_D+  UGGGGCGUGGUGCAAU---GGC--AGCACACCAGACU-UUG-GAUCUGUGGA----UCUUGGUUCGAAUCCAGGCGCCCCAG
ATM_20_NS_D+  UGGGGGCUCGUCUAAU---GGC--AGGACGACAGACU-CUG-GAUCUGUUUG----UAGAGGUUCGAAUCCUUUGCCCCCAG
RBA_10_pl_D+  UACCCCGUGGUGUAAUU--GGC--AACACAGCUGGUU-UUG-GUCCAGCCAU----UCUAGGUUCGAGUCCUAGCGGGGUAG
RBA_2A_pl_D+  GCCGGAGUAGCAGUUGUU-GGUC-GCUGCACCUGACU-CUG-AAUCAGGAGUU---CAUUGGUUCGAUUCCAAUCUCCGGUG
RBA_2B_pl_D+  GCCGGAGUAGCAGUUGUU-GGUC-GCUGCACCUCGCU-CUG-AACGAGGAGUCCAUUGGUUCAAUUCCUGUCCUCGGCCCUG
PHM_10_pl_D+  AGGCCCAUGGUGUAAU---GGC--AGCACAGCUGUUU-UUG-GUGCAGCUCG----UCGGGGUUCGAAUCCCUGUGGGCCUA
PLM_10_pl_D+  UCGGAUGUCGGCUAAU---GGU--AAGCCAACUGCCU-UUG-AAGCAGUGGUA---UGAAGGUUCAACUCCUUCCGCCCGGG
PLM_10_pl_D+  UACCCCGUAGUGUAAC---GGU--AGCACAGCAGGUU-UUG-GUCCUGCACG----UUCAGGUUCGAAUCCUGGCGGGGUAA
PHM_20_pl_D+  AGGCCCAUGGUGUAAC---GGU--AGCACUAAAGAUU-CUG-AUUCUUUCAG----UCGGGGUUCGAAUCCCUGUGGGCCUA
PLM_20_pl_D+  UGCCUGAUGGUGUAAU---GGU--AGCACGAGUGACU-CUG-GAUCACUUAG----UUGAGGUUCGAAUCCUCAUCGGGCAA
CAA_10_ve_D+  AGGCCGAUGGUGUAAU---GGU--AACACUACUGGUU-UUG-GUCCAGUCAU----UUGGGGUUCGAGUCCCUAUCGGCCUG
OTE_10_ve_D+  UGCCCGAUGGUGUAAU---GGC--AGCACAGGCGACU-UUG-ACUCGCCUAG----UCAUGGUUCGAAUCCAUGUCGGGCAG
MIN_10_ve_D+  UGCCCGGUGGUGUAAU---GGU--AGCACAGAGGUCU-UUG-GAGCCUUUAG----UCAUGGUUCGAAUCCAUGCCGGGCAG
AMU_10_ve_D+  AGCCCCAUAGUGUAAUU--GGU--AACACACCUGAUU-UUG-GUUCAGUAUU----UCUAGGUUCGAGUCCUGGUGGGGCUG
OTE_20_ve_D+  UGCCCGAUGGUGUAAU---GGU--AGCACAAACGACU-CUG-ACUCGUUUUG----UCUAGGUUCGAGUCCUAGUCGGGCAG
MIN_20_ve_D+  UGCCGCGUGGUGCAAUU--GGU--AGCACAUCAGACU-CUG-GAUCUGAGUGU---UCUAGGUUCGAAUCCUAGCGCGGCAG
AMU_20_ve_D+  AGCCCUAUAGUGUAAUC--GGU--AACACAGCGGAUU-CUG-GUUCCGUAUU----UUGGGGUUCGAGUCCUCAUAGGGCUA
IAL_10_gs_D+  UGCCGGGUCGUCUAAU---GGU--AGGACAGCGGCCU-UUG-GAGCCGUAUG----UAGAGGUUCGAAUCCUCUCCCGGCAG
IAL_20_gs_D+  UGUCCCGUGGUGUAACU--GGC--AACACGUCUGACU-CUG-GAUCAGAAGAG---UCCAGGUUCGAGCCCUGGCGGGACAA
SSM_10_sp_D+  UGGGGCGUUGGCAAGC---GGUU-AAGCCACCGGUUU-UUG-GAGCCGGUAUU---CGCAGGUUCGAAUCCUGCCGCCCCAG
```

```
                 12345678901234567--890ab1234567890123-456-78901234567---890123456789012345678901 23
SSM_20_sp_D+  UGGCCGAUAGUGUAAU---GGU--AGCACAACAGAUU-CUG-GAUCUGUUUG----UGAGGGUUCAAAUCCUUCUCGGCCAG
PML_10_te_D-  AGGCCCAUAGCCAAGU---GGU--AAGGCAACGGACU-UUG-ACUCCGUGAUU---CGUUGGUUCAAAUCCAGCUGGGCCUG
ACL_10_te_D-  AGGCCCAUAGCCAAGC---GGU--AAGGCAACGGACU-UUG-ACUCCGUCACU---CGUAGGUUCAAAUCCUGCUGGGCCUG
POY_10_te_D-  AGGCCCAUAGCCAAGU---GGU--AAGGCAACGGACU-UUG-ACUCCGUGAUU---CGUUGGUUCAAAUCCAGCUGGGUCUG
CTH_10_fi_D-  AGGGAUGUAGCCAAGC---GGC--AAGGCACCAGACU-UUG-ACUCUGGCAUUU--CGUAGGUUCGAAUCCUGCCAUCCCUG
AOE_10_fi_D-  UGGGAUAUAGCCAAGUC--GGU--AAGGCAACGGACU-UUG-ACUCCGUCAUUC--CGCAGGUUCGAGUCCUGCUAUCCCAG
CTH_20_fi_D-  UGCGGGAUGGUGUAAG---GGU--AGCACAAAUGACU-CUG-GAUCAUUGUG----UGAGGGUUCGAAUCCUUCUCCCGCAG
AYM_10_ac_D+  UCCGGGGUCGUCUAAC---GGU--AAGACAGCGGUUU-UUG-GUGCCGAGAA----UAGGGGUUCGAUUCCUCUCCCCGGAA
NDE_10_ht_D+  UGGGGGAUCGUCUAGC---GGU--AGGACGCCAGCCU-UUG-GAGCUGGCUA----CCUAGGUUCGAUUCCUAGUCCCCCAG
NDE_20_ht_D+  UGGGGGAUCGUCUAGC---GGU--AGGACGUCAGUCU-CUG-GAUCUGACUA----CCUAGGUUCGAUUCCUAGUCCCCCAG
TTH_10_dt_D+  UGGGGCGUCGUCUAAC---GGC--AGGACAGCGGACU-UUG-GAUCCGCCGG----UGGUGGUUCGAGUCCACCCGCCCCAG
DRA_10_dt_D+  AGGGAUAUGGUGUAAU---GGU--AGCACAACAGAUU-UUG-GUUCUGUUUG----UCUAGGUUCGAAUCCUUGUAUCCCUG
MRB_10_dt_D+  UGGGGCAUCGUCUAAU---GGC--AGGACAACGGUCU-UUG-GAACCGUCGG----UCGUGGUUCGAGUCCACGUGCCCCAG
MHD_10_dt_D+  UGGGGCGUCGUCUAAU---GGC--AGGACAGCGGACU-UUG-GAUCCGCCGG----UCGUGGUUCGAGUCCACGCGCCCCAG
OPR_10_dt_D+  UGGGGCGUCGUCUAAG---GGU--AGGACAGCGGUCU-UUG-GAACCGCCGG----UCGUGGUUCGAAUCCACGCGCCCCAG
TRA_10_dt_D+  UGGGGUGUCGUCUAAU---GGU--AGGACCGCGGAUU-UUG-GUUCCGUUAA----UCGGGGUUCGAGUCCCUGCACCCCAG
TTH_20_dt_D+  UGGGGUGUCGUCUAAG---GGU--AGGACAGCGGACU-CUG-GAUCCGCCGG----UCGUGGUUCGAAUCCACGCACCCCAG
DRA_20_dt_D+  UGGGGUAUCGUCUAAU---GGC--AGGACGUCGGUUU-CUG-GCACCGUUAA----UCAAGGUUCGAGUCCUUGUACCCCAG
MRB_20_dt_D+  UGGGGCAUCGUCUAAC---GGC--AGGACUACGGAUU-CUG-GCUCCGUCAA----UCGUGGUUCGAAUCCACGUGCCCCAG
MHD_20_dt_D+  UGGGGCGUCGUCUAAU---GGC--AGGACAGCGGACU-CUG-GAUCCGCCGG----UCGUGGUUCGAGUCCACGCGCCCCAG
OPR_20_dt_D+  UGGGGCAUCGUCUAAC---GGC--AGGACGGCGGACU-CUG-GAUCCGUUGG----UCGUGGUUCGAAUCCACGUGCCCCAG
TRA_20_dt_D+  UCAGGAAUGGUGUAAC---GGU--AGCACGACGCACU-CUG-GAUGCGUUAG----UCCUGGUUCGAAUCCAGGUUCCUGAG
GFO_10_ba_D-  UGGCCUAUGGUGUAACU--GGC--AACACGUCUGGUU-UUG-GUCCAGAAGAG---UCUAGGUUCGAGCCCUAGUAGGCCAA
FJO_10_ba_D-  UGGUCUAUGGUGUAAU---GGU--AACACUACGGUUU-UUG-GUGCCGUCUU----UCUAGGUUCGAGUCCUAGUAGACCAA
PGI_10_ba_D-  UGGUCUCAUGGUGUAAU--GGU--AGCACAACAGGUU-UUG-GUUCUGUUUG----UCAAGGUUCGAAUCCUUGUGAGACAA
BFR_10_ba_D-  UGUCCUAUGGUGUAAU---GGU--AGCACAACAGUUU-UUG-GUUCUGUUUG----UCUAAGUUCGAAUCUUGGUAGGACAA
PDI_10_ba_D-  UGUCUCAUGGUGUAAU---GGU--AGCACUACAGUUU-UUG-GUUCUGUCUG----UCGAGGUUCGAAUCCUCGUGAGACAA
SMG_10_ba_D-  AGACUCGUUGUGUAAU---GGU--AGCACAGCAGAUU-UUG-GUUCUGUUAG----UUGGGGUUCAAAUCCCUACGGGUCUG
COC_10_ba_D-  UGGUCUAUGGUGUAAU---GGU--AACACAGCAGAUU-UUG-GUUCUGUUAU----UCUAGGUUCGAGUCCUAGUAGACCAA
RAN_10_ba_D-  AGACCCAUGGUGUAAUU--GGC--AACACAGCAGAUU-UUG-GUUCUGUUAU----UCGGGGUUCGAGUCCCUGUGGGUCUA
PPN_10_ba_D-  UGUCUCAUGGUGUAAU---GGU--AGCACUGCAGUUU-UUG-GUUCUGCCUG----UCCAAGUUCGAAUCUUGGUGAGACAA
BBL_10_ba_D-  UAGACCCGUUGUGUAAC--GGU--AGCACAGCAGAUU-UUG-GUUCUGUUAG----UUGGGGUUCGAGUCCCUACGGGUCUG
CAO_10_ba_D-  UGGUCCAUGGUGUAAUU--GGC--AACACUCCGGUUU-UUG-GUACCGUCAU----UCAAGGUUCGAGUCCUUGUGGACCAA
ZGA_10_ba_D-  UGGCCUAUGGUGUAAUU--GGC--AACACAGCUGGUU-UUG-GUCCAGUCGU----UCUAGGUUCGAGUCCUAGUAGGCCAA
SRU_10_ba_D+  UGCCCGGUGGUCUAAU---GGC--AGGACAGCGGCCU-UUG-GAGCCGUUAG----UGGUGGUUCGAAUCCACCCCGGGCAA
PGI_20_ba_D-  UGUCUCAUGGUGUAAU---GGU--AGCACAACAGAUU-CUG-GUCCUGUUUG----UCAAGGUUCGAAUCCUUGUGAGACAA
BFR_20_ba_D-  UGGGCUAUGGUGUAAU---GGU--AACACUACAGAUU-CUG-GUCCUGUCAU----UUCUGGUUCGAGUCCAGAUAGCCCAA
PDI_20_ba_D-  UGUUCUAUGGUGUAAU---GGU--AGCACAACAGAUU-CUG-GUCCUGUUUG----UCCUGGUUCGAAUCCAGGUAGAACAA
PPN_20_ba_D-  UGUCUUAUGGUGUAAU---GGU--AGCACUGCAGUUU-CUG-GAUCUGCCUG----UCUAGGUUCGAAUCCUAGUAAGACAA
SRU_20_ba_D+  UGCCCAGUAGCUCAAUC--GGC--AGAGCGUCCGGCU-CUG-GACCGGGAGGC---UGGUGGUUCGAAUCCACCCUGGGCAA
```

Multiple-aligned tRNA^{Gln} sequences from GlnRS-containing bacteria. Each entry is annotated by a three-letter code for the organism (see Additional files 1 and 2), followed by a numeral (1 indicating isoacceptor 1 and 2 indicating isoacceptor 2), a letter (A and B stand for two isoforms for the same isoacceptor while 0 indicates that there is only one isoform) and a two-letter code indicating the phylum (see Table 1 of main text). The canonical numbering of tRNA nucleotides is shown at the first line in each new page (colored red).
