# Supplementary material for: Evolutionary insights about bacterial GlxRS from whole genome analyses: is GluRS2 a chimera?
Source: BMC Evol Biol. 2014 Feb 12;14:26. doi: 10.1186/1471-2148-14-26 (PMC3927822; doi:10.1186/1471-2148-14-26)
Supplement: Additional file 8 — Identity features of tRNAGln isotypes (tRNAGln1 and tRNAGln2) at the nucleotides 32, 38 and 37 of the anticodon loop in bacterial genomes with GlnRS gene. [file 1471-2148-14-26-S8.pdf]

Identity features of tRNA^{Gln} isotypes (tRNA^{Gln1} and tRNA^{Gln2}) at the nucleotides 32, 38 and 37 of the anticodon loop in bacterial genomes with GlnRS gene.*

Bacteria names	*class/phylum*	tRNA^{Gln1}	tRNA^{Gln2}
Wigglesworthia glossinidia	γ	U-U-A	
Enterobacter sp	γ	U-U-A	U-U-A
Cronobacter sakazakii	γ	U-U-A	U-U-A
Citrobacter rodentium	γ	U-U-A	U-U-A
Proteus mirabilis	γ	U-U-A	
Edwardsiella ictaluri	γ	U-U-A	U-U-A
Candidatus Hamiltonella defensa	γ	U-U-A	U-U-A
Xenorhabdus bovienii	γ	U-U-A	U-U-A
Candidatus Riesia pediculicola	γ	U-U-A	
Rahnella sp. Y9602	γ	U-U-A	U-U-A
Candidatus Moranella endobia	γ	U-U-A	U-U-A
Haemophilus somnus 129PT	γ	U-U-A	
Gallibacterium anatis	γ	U-U-A	U-U-A
Stenotrophomonas maltophilia	γ	U-U-A	C-C-A
Pseudoxanthomonas suwonensis	γ	U-U-A	C-C-A
Vibrio anguillarum	γ	U-U-A	
Alteromonas macleodii	γ	U-U-A	
Ferrimonas balearica	γ	U-U-A	
Kangiella koreensis	γ	U-U-A	
Actinobacillus pleuropneumoniae	γ	U-U-A	
Aeromonas hydrophila	γ	U-U-A	
Baumannia cicadellinicola	γ	U-U-A	U-U-G
Buchnera aphidicola	γ	U-U-A	
Candidatus Blochmannia floridanus	γ	U-U-A	U-U-G
Colwellia psychrerythraea	γ	U-U-A	
Escherichia coli	γ	U-U-A	U-U-A
Haemophilus ducreyi	γ	U-U-A	
Idiomarina loihiensis	γ	U-U-A	
Mannheimia succiniciproducens	γ	U-U-A	
Pasteurella multocida	γ	U-U-A	
Photorhabdus luminescens	γ	U-U-A	U-U-A
Photobacterium profundum	γ	U-U-A	
Pseudoalteromonas atlantica	γ	U-U-A	
Psychromonas ingrahamii	γ	U-U-A	
Salmonella enterica	γ	U-U-A	
Shigella dysenteriae	γ	U-U-A	U-U-A
Sodalis glossinidius	γ	U-U-A	U-U-A
Vibrio parahaemolyticus	γ	U-U-A	
Xanthomonas oryzae	γ	U-U-A	C-C-A
Xylella fastidiosa	γ	U-U-A	C-C-A
Yersinia pseudotuberculosis	γ	U-U-A	U-U-A
Klebsiella pneumoniae	γ	U-U-A	U-U-A
Dickeya dadantii	γ	U-U-A	U-U-A
Pantoea ananatis	γ	U-U-A	U-U-A
Glaciecola sp.	γ	U-U-A	
Methylomonas methanica	γ	U-U-A	
Methylomicrobium alcaliphilum	γ	U-U-A	
Francisella tularensis	γ	U-U-A	
Thioalkalimicrobium cyclicum	γ	U-U-A	
Allochromatium vinosum	γ	U-U-A	C-C-A
Halothiobacillus neapolitanus	γ	U-U-A	

Tolumonas auensis	γ	U-U-A	U-U-A
Dichelobacter nodosus	γ	U-U-A	
Candidatus Ruthia magnifica	γ	U-U-A	
Candidatus Vesicomyosocius okutanii	γ	U-A-A	
Legionella pneumophila	γ	U-U-A	
Thiomicrospira crunogena	γ	U-U-A	
Serratia proteamaculans	γ	U-U-A	U-U-A
Pseudomonas aeruginosa	γ	U-U-A	
Saccharophagus degradans	γ	U-U-A	
Psychrobacter arcticum	γ	U-U-A	
Hahella chejuensis	γ	U-U-A	
Cellvibrio japonicus	γ	U-U-A	
Azotobacter vinelandii	γ	U-U-A	
Acinetobacter sp.	γ	U-U-A	
Moraxella catarrhalis	γ	U-U-A	
Marinobacter aquaeolei	γ	U-U-A	
Chromohalobacter salexigens	γ	U-U-G	U-U-G
Halomonas elongata	γ	U-U-G	
Alcanivorax borkumensis	γ	U-U-A	
Teredinibacter turnerae	γ	U-U-A	
Marinomonas sp.	γ	U-U-A	
Gamma proteobacterium HdN1	γ	U-U-A	
Neisseria meningitidis	β	U-U-A	
Laribacter hongkongensis	β	U-U-A	
Pseudogulbenkiania sp	β	U-U-A	
Burkholderia mallei	β	U-U-A	
Polynucleobacter sp	β	U-U-A	
Achromobacter xylosoxidans	β	U-U-A	
Taylorella equigenitalis	β	U-U-A	
Pusillimonas sp	β	U-U-A	
Polaromonas sp	β	U-U-A	
Acidovorax avenae	β	U-U-A	
Verminephrobacter eiseniae	β	U-U-A	
Delftia acidovorans	β	U-U-A	
Variovorax paradoxus	β	U-U-A	
Comamonas testosteroni	β	U-U-A	
Alicycliphilus denitrificans	β	U-U-A	
Ramlibacter tataouinensis	β	U-U-A	
Methylibium petroleiphilum	β	U-U-A	
Herminiimonas arsenicoxydans	β	U-U-A	
Minibacterium massiliensis	β	U-U-A	
Herbaspirillum seropedicae	β	U-U-A	
Candidatus Zinderia insecticola	β	U-U-A	
Collimonas fungivorans	β	U-U-A	
Leptothrix cholodnii	β	U-U-A	
Thiomonas intermedia	β	U-U-A	
Nitrosomonas europaea	β	U-U-A	
Aromatoleum aromaticum	β	U-U-A	
Azoarcus sp	β	U-U-A	
Thauera sp	β	U-U-A	
Methylotenera mobilis	β	U-U-A	
Methylovorus glucosetrophus	β	U-U-A	
Accumulibacter phosphatis	β	U-U-A	
Sideroxydans lithotrophicus	β	U-U-A	
Gallionella capsiferriformans	β	U-U-A	
Bordetella bronchiseptica	β	U-U-A	
Chromobacterium violaceum	β	U-U-A	

Dechloromonas aromatica	*β*	U-U-A	
Methylobacillus flagellatus	*β*	U-U-A	
Nitrosospira multiformis	*β*	U-U-A	
Ralstonia solanacearum	*β*	U-U-A	
Rhodoferax ferrireducens	*β*	U-U-A	
Thiobacillus denitrificans	*β*	U-U-A	
Variovorax paradoxus EPS	*β*	U-U-A	
Cupriavidus metallidurans	*β*	U-U-A	
Lawsonia intracellularis	*δ*	U-U-G	U-C-A
Anaeromyxobacter sp	*δ*	C-A-G	C-A-G
Desulfovibrio vulgaris	*δ*	U-U-G	U-C-G
Myxococcus xanthus	*δ*	C-C-A	C-C-A
Pelobacter carbinolicus	*δ*	U-U-A	C-C-A
Anaeromyxobacter dehalogenans	*δ*	C-A-G	C-A-G
Geobacter lovleyi	*δ*	U-U-A	
Hippea maritima	*δ*	C-A-G	C-A-G
Desulfarculus baarsii	*δ*	C-A-G	U-C-G
Syntrophobacter fumaroxidans	*δ*	C-A-G	C-A-G
Haliangium ochraceum	*δ*	U-U-G	C-A-G
Stigmatella aurantiaca	*δ*	C-A-A	C-C-A
Syntrophus aciditrophicus	*δ*	U-U-G	C-C-A
Desulfobacca acetoxidans	*δ*	C-A-G	C-A-G
Desulfobacterium autotrophicum	*δ*	U-U-A	C-C-A
Bdellovibrio bacteriovorus	*δ*	U-U-A	
Desulfohalobium retbaense	*δ*	U-U-G	U-U-G
Desulfotalea psychrophila	*δ*	U-U-A	
Desulfurivibrio alkaliphilus	*δ*	C-C-A	C-C-A
Desulfobulbus propionicus	*δ*	U-U-A	C-C-A
Candidatus Desulfococcus oleovorans	*δ*	U-U-A	U-C-G
Desulfauibacillum Alkenivorans	*δ*	U-U-G	C-A-G
Desulfomicrobium baculatum	*δ*	U-U-G	C-C-A
Oligotropha carboxidovorans	*α*	U-U-A	C-C-A
Nitrobacter hamburgensis	*α*	U-U-A	C-C-A
Bradyrhizobium japonicum	*α*	U-U-A	C-C-A
Rhodopseudomonas palustris	*α*	U-U-A	C-C-A
Mesorhizobium sp. BNC1	*α*	U-U-G	C-C-A
Mesorhizobium loti	*α*	C-C-A	C-C-A
Sulfurimonas denitrificans	*ε*	U-U-G	
Arcobacter butzleri	*ε*	U-U-G	
Sulfuricurvum kujiense	*ε*	U-U-G	
Sulfurospirillum deleyianum	*ε*	U-U-G	
Sulfurovum sp.	*ε*	U-U-G	
Nitratifractor salsuginis	*ε*	U-U-G	
Spirochaeta smaragdinae	*sp*	U-A-G	U-A-G
Solibacter usitatus	*ad*	C-A-G	C-A-G
Opitutus terrae	ve	C-C-A	C-C-A
Coraliomargarita akajimensis	ve	U-U-G	
Methylacidiphilum infernorum	ve	C-A-G	C-A-G
Akkermansia muciniphila	ve	U-U-G	U-U-G
Planctomyces limnophilus	*pl*	C-A-A	C-A-G
Phycisphaera mikurensis	*pl*	U-U-G	U-U-A
Rhodopirellula baltica	*pl*	U-U-G	C-A-A
Ignavibacterium album	*gs*	C-A-G	C-A-G
Anaerolinea thermophila	*ns*	C-A-G	C-A-G
Clostridium thermocellum	*fi*	C-C-A	C-A-G
Alkaliphilus oremlandii	*fi*	C-C-A	
Clostridium clariflavum	*fi*	C-C-A	C-A-G

Desulfotomaculum reducens	*fi*	C-C-A	C-C-A
Desulfotomaculum ruminis	*fi*	C-A-G	C-A-G
Clostridium perfringens	*fi*	C-C-A	C-A-G
Clostridium ljungdahlii	*fi*	C-C-A	C-A-G
Bacillus pseudofirmus	*fi*	C-C-A	
Bacillus cellulosilyticus	*fi*	C-C-A	
Bacillus selenitireducens	*fi*	C-C-A	
Acholeplasma laidlawii	*te*	C-C-A	
Candidatus Phytoplasma mali	*te*	C-C-A	
Phytoplasma OY	*te*	C-C-A	
Thermus thermophilus	*dt*	C-A-G	C-A-G
Deinococcus radiodurans	*dt*	U-U-G	U-C-G
Meiothermus ruber	*dt*	C-A-G	U-C-G
Truepera radiovictrix	*dt*	U-U-G	C-A-G
Oceanithermus profundus	*dt*	C-A-G	C-A-G
Marinithermus hydrothermalis	*dt*	C-A-G	C-A-G
Salinibacter ruber	*ba*	C-A-G	C-A-G
Gramella forsetii	*ba*	U-U-G	
Flavobacterium johnsoniae	*ba*	U-U-G	
Porphyromonas gingivalis	*ba*	U-U-G	U-U-G
Bacteroides fragilis	*ba*	U-U-G	U-U-G
Parabacteroides distasonis	*ba*	U-U-G	U-U-G
Candidatus Sulcia muelleri	*ba*	U-U-G	
Capnocytophaga ochracea	*ba*	U-U-G	
Riemerella anatipestifer	*ba*	U-U-G	
Paludibacter propionicigenes	*ba*	U-U-G	U-A-G
Blattabacterium sp.	*ba*	U-U-G	
Cellulophaga algicola	*ba*	U-U-G	
Zobellia galactanivorans	*ba*	U-U-G	
Candidatus Nitrospira defluvii	*ht*	C-A-G	C-A-G
Acidimicrobidae bacterium	*ac*	U-U-G	U-U-G

* see Table 1 (main text) for class/phylum abbreviation.
